# SerpinB3 Differently Up-Regulates Hypoxia Inducible Factors-1α and -2α in Hepatocellular Carcinoma: Mechanisms Revealing Novel Potential Therapeutic Targets

**DOI:** 10.3390/cancers11121933

**Published:** 2019-12-04

**Authors:** Stefania Cannito, Beatrice Foglia, Gianmarco Villano, Cristian Turato, Teresa C Delgado, Elisabetta Morello, Fabrizio Pin, Erica Novo, Lucia Napione, Santina Quarta, Mariagrazia Ruvoletto, Silvano Fasolato, Giacomo Zanus, Sebastiano Colombatto, Fernando Lopitz-Otsoa, David Fernández-Ramos, Federico Bussolino, Salvatore Sutti, Emanuele Albano, Maria Luz Martínez-Chantar, Patrizia Pontisso, Maurizio Parola

**Affiliations:** 1 Department of Clinical and Biological Sciences, Unit of Experimental Medicine & Clinical Pathology, University of Torino, 10125 Torino, Italy; stefania.cannito@unito.it (S.C.); beatrice.foglia@unito.it (B.F.); elisabetta.morello@unito.it (E.M.); fpin@iu.edu (F.P.); erica.novo@unito.it (E.N.); 2 Department of Surgery, Oncology and Gastroenterology, University of Padova, 35128 Padova, Italy; gianmarco.villano@unipd.it (G.V.); mariagrazia.ruvoletto@unipd.it (M.R.); silvano.fasolato7@gmail.com (S.F.); 3 Veneto Institute of Oncology IOV—IRCCS, 35128 Padova, Italy; cristianturato@gmail.com; 4 Liver Disease and Metabolism Laboratory, CIC bioGUNE, Centro de Investigacion Biomedica en Red de Enfermedades Hepaticas y Digestivas (Ciberehd), Technology Park of Bizkaia, 48160 Derio, Bizkaia, Spain; tcardoso@cicbiogune.es (T.C.D.); flopitz@cicbiogune.es (F.L.-O.); dfernandez.ciberehd@cicbiogune.es (D.F.-R.); mlmartinez@cicbiogune.es (M.L.M.-C.); 5 Department of Applied Science and Technology, Politecnico di Torino, 10129 Torino, Italy; lucia.napione@ircc.it; 6 Laboratory of Vascular Oncology Candiolo Cancer Institute—FPO IRCCS (Istituto di Ricovero e Cura a Carattere Scientifico), 10060 Candiolo, Italy; federico.bussolino@ircc.it; 7 Department of Medicine, University of Padova, 35128 Padova, Italy; santina.quarta@unipd.it (S.Q.); patrizia@unipd.it (P.P.); 8 Hepatobiliary Surgery, University of Padova, 35128 Padova, Italy; giacomo.zanus@unipd.it; 9 Department of Oncology, University of Torino, 10125 Torino, Italy; sebastiano.colombatto@unito.it; 10 Department of Health Sciences and Interdisciplinary Research Center for Autoimmune Diseases, University Amedeo Avogadro of East Piedmont, 28100 Novara, Italy; salvatore.sutti@med.uniupo.it (S.S.); emanuele.albano@med.uniupo.it (E.A.)

**Keywords:** SerpinB3, hypoxia, hypoxia inducible factors HIF-1α and HIF-2α, HCC, NEDD8, NEDDylation

## Abstract

Background: SerpinB3 (SB3) is a hypoxia and hypoxia-inducible factor (HIF)-2α-dependent cysteine-protease inhibitor up-regulated in hepatocellular carcinoma (HCC), released by cancer cells and able to stimulate proliferation and epithelial-to-mesenchymal-transition. Methods: In the study we employed transgenic and knock out SerpinB3 mice, liver cancer cell line, human HCC specimens, and mice receiving diethyl-nitrosamine (DEN) administration plus choline-deficient L-amino acid refined (CDAA) diet (DEN/CDAA protocol). Results: We provide detailed and mechanistic evidence that SB3 can act as a paracrine mediator able to affect the behavior of surrounding cells by differentially up-regulating, in normoxic conditions, HIF-1α and HIF-2α. SB3 acts by (i) up-regulating HIF-1α transcription, facilitating cell survival in a harsh microenvironment and promoting angiogenesis, (ii) increasing HIF-2α stabilization via direct/selective NEDDylation, promoting proliferation of liver cancer cells, and favoring HCC progression. Moreover (iii) the highest levels of NEDD8-E1 activating enzyme (NAE1) mRNA were detected in a subclass of HCC patients expressing the highest levels of HIF-2α transcripts; (iv) mice undergoing DEN/CDAA carcinogenic protocol showed a positive correlation between SB3 and HIF-2α transcripts with the highest levels of NAE1 mRNA detected in nodules expressing the highest levels of HIF-2α transcripts. Conclusions: These data outline either HIF-2α and NEDDylation as two novel putative therapeutic targets to interfere with the procarcinogenic role of SerpinB3 in the development of HCC.

## 1. Introduction

SerpinB3 (SB3) is a serine protease inhibitor up-regulated in several malignancies of epithelial origin [1,2], including hepatocellular carcinoma (HCC) [1,3]. SB3, virtually undetectable in normal hepatocytes, is up-regulated in cirrhotic livers, dysplastic nodules, HCC [1,2,3,4] and hepatoblastoma [5], and proposed to be involved in early carcinogenesis [6]. In liver cancer cells, by acting as an autocrine and/or paracrine mediator, SB3 induces apoptosis resistance [7], cell proliferation [8], epithelial-to-mesenchymal transition (EMT), and increased invasiveness [9,10]. Moreover, SB3 is up-regulated by oncogenic Ras and promotes NF-kB-dependent expression of inflammatory cytokines, autocrine IL-6 signaling [10,11] as well as c-Myc expression through the Yap pathway [12], all events potentially favoring tumor progression. In addition, SB3 transcription, synthesis, and extracellular release are up-regulated by hypoxia through selective HIF-2α-dependent and redox-sensitive mechanisms in liver cancer cells, with a relevant positive correlation between HIF-2α and SB3 transcript levels in human HCC specimens, related to early recurrence [13].

Along these lines, tumor hypoxia is considered as an independent negative prognostic indicator with a significant risk for the patient to develop metastasis that may escape therapy [14,15]. Hypoxic microenvironment is also believed to contribute to cancer progression by activating adaptive transcriptional programs involved in promoting angiogenesis, EMT, and increased invasiveness in human epithelial cancer cells [16,17,18,19]. In this scenario, hypoxia-inducible factor (HIF)-1α and HIF-2α, exhibit both common and unique patterns of downstream target genes that may exert distinct roles in tumorigenesis. HIF-2α, at least in some tumors, shows a greater oncogenic capacity than HIF-1α, and its overexpression correlates with poor patient outcome in colorectal carcinoma, melanoma, ovarian cancer, non-small cell lung cancers, and HCC [20,21]. Accordingly, HIF-2α (not HIF-1α) can favor proliferation of cancer cells by promoting c-Myc activity, and radio- and chemo-resistance, self-renewal capability, and stem cell phenotype in non-stem cell populations via Oct-4, Nanog, and c-Myc as well as by contributing to the maintenance of cancer stem cell (CSCs) properties [19,20]. By contrast, HIF-1α preferentially plays a role in reprogramming cancer metabolism by activating the transcription of glycolytic enzyme genes [21].

In the present study we propose that SB3 can affect the behavior of liver cancer cells by increasing both HIF-1α, at transcription level through a redox-dependent mechanism, and HIF-2α protein levels by direct and selective NEDDylation/stabilization of HIF-2α, then resulting in up-regulation of related target genes. These data outline either HIF-2α and NEDDylation as two novel putative therapeutic targets to interfere with the pro-carcinogenic role of SerpinB3 in the development of HCC.

## 2. Results

### 2.1. SerpinB3 Overexpression Up-Regulates HIF-1α and HIF-2α Hepatic Protein Levels

**Figure 1:**
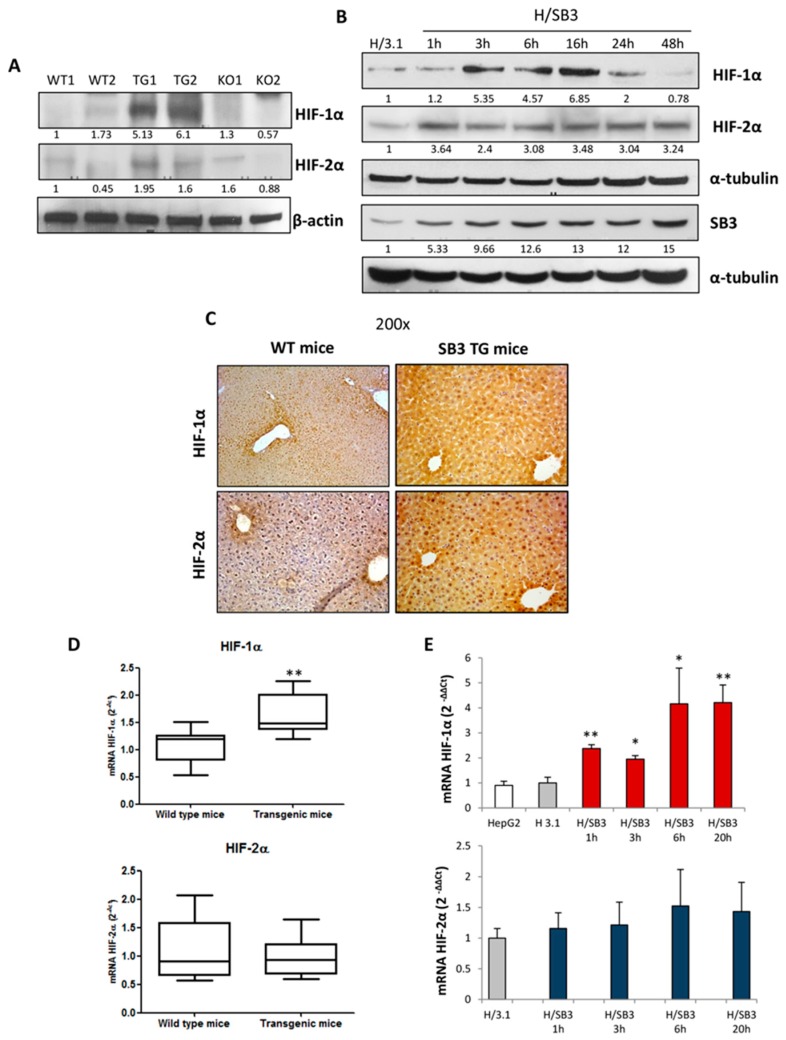
Analysis of hypoxia-inducible factor (HIF)-1α and HIF-2α transcript and protein levels in SerpinB3 (SB3) transgenic mice and SB3 overexpressing HepG2 cells. (**A**) Western blot analysis of HIF-1α and HIF-2α in total extracts of WT mice (WT1—C57Bl6J mice, control for TG mice; WT2—Balb-C mice, control for knock out (KO) mice), SB3-TG mice (TG 1,2), and knock-out mice for SB3 (KO 1,2); equal loading was evaluated by re-probing membranes for β-actin. BIORAD Quantity One software was used to perform the densitometric analysis (data are expressed as Fold Change relative to the normalized control condition expression). (**B**) Western blot analysis of HIF-1α, SB3, and HIF-2α in total extracts obtained from H/3.1 and H/SB3 at indicated time points. Cells were seeded in normoxic conditions to obtain the desired sub-confluence level (65%–70%) and after 20 h from plating time course analysis was performed from 1 h up to 48 h; equal loading was evaluated by re-probing membranes for α-tubulin. BIORAD Quantity One software was used to perform the densitometric analysis (data are expressed as Fold Change relative to the normalized control condition expression). (**C**) IHC analysis for HIF-1α and HIF-2α performed on liver sections from wild-type (WT) and SB3-TG (TG) mice. Original magnification as indicated. (**D**) qPCR analysis of HIF-1α and HIF-2α in WT and TG mice. Data are expressed as means ± SEM of three independent experiments (** *p* < 0.01 vs. WT littermates). (**E**) qPCR analysis of HIF-1α and HIF-2α transcripts in control HepG2 cells, HepG2 cells transfected with empty vector pCDNA3.1 (H/3.1), and HepG2 cells over-expressing SB3 (H/SB3). Data are expressed as means ± SEM of three independent experiments (* *p* < 0.05 or ** *p* < 0.01 vs. H/3.1).

In preliminary experiments we evaluated HIF-α subunits protein levels in total extracts obtained from the liver of WT, SB3 transgenic—(TG) mice, or SB3 knock out (KO) mice, as well as in in vitro experiments performed under normoxic conditions in HepG2 cells over-expressing SB3 (H/SB3) or transfected with an empty vector (H/3.1). HIF-1α and HIF-2α protein were almost absent in SB3-KO mice, while were strongly up-regulated in the liver extracts from SB3-TG mice or H/SB3 cells (Figure 1A,B). In H/SB3 cells HIF-1α over-expression was evident within 24 h and then declined with the time, whereas HIF-2α levels were up-regulated, in a sustained manner throughout the entire time course. Accordingly, SB3-TG mice were characterized by a marked and diffuse increase of hepatic HIF-1α and HIF-2α immune-positivity in hepatocyte cytoplasm and nuclei (Figure 1C) whereas HIF-1α and HIF-2α positive staining in wild tipe WT mice was limited to few hepatocytes around centrilobular vein. However, the analysis of HIFs transcripts in vivo and in vitro showed that only HIF-1α mRNA were significantly increased, while HIF-2α up-regulation did not involve the transcriptional levels (Figure 1D,E), suggesting a post-translational modifications in SB3-dependent modulation of HIF-2α.

### 2.2. Up-Regulation of HIF-1α by SerpinB3 Is Related to Intracellular Generation of ROS

**Figure 2:**
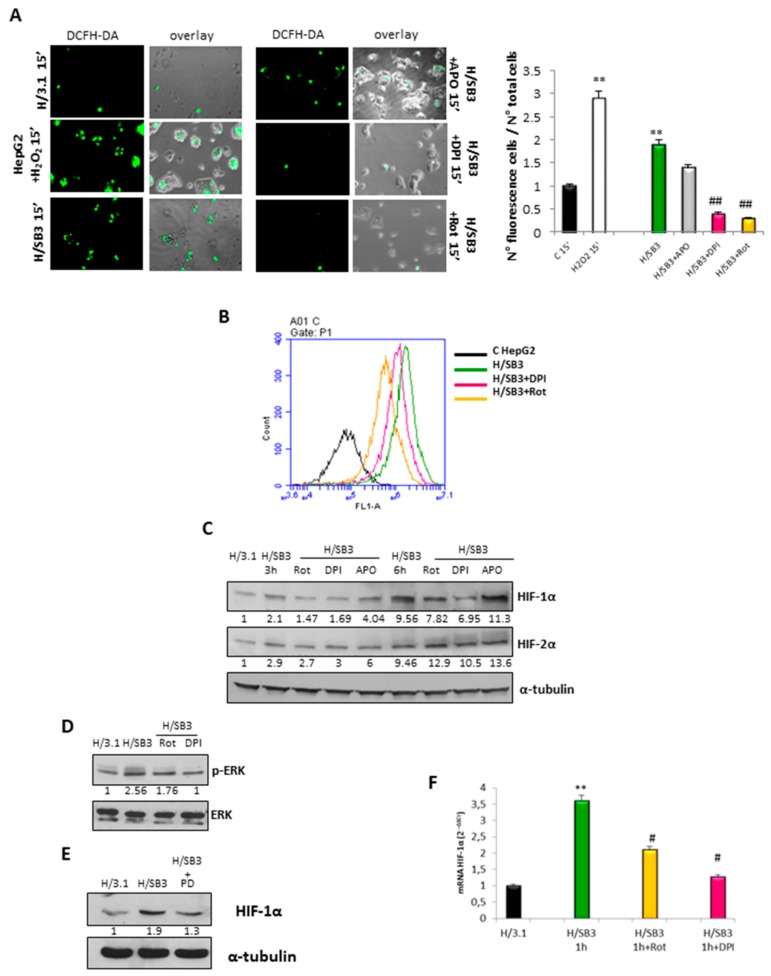
Induction and stabilization of HIF-1α by SerpinB3-dependent up-regulation of intracellular ROS generation by mitochondria. (**A**) Detection and quantification of intracellular ROS (DCFH-DA probe) in control H/3.1 cells or H/SB3 cells by using morphological analysis with florescence microscope. Graph of quantification of ROS positive cells represents the mean number of cells per microscope field ± SD of three different experiments (** *p* < 0.01 vs. control condition and ## *p* < 0.01 vs. related H/SB3). In same experiments H/SB3 cells were pretreated with Rotenone (Rot, 2.5 µM), diphenyleneiodonium (DPI) (1 µM), or apocynin (APO, 250 µM). H_2_O_2_ 50 µM was used as positive control. (**B**) Quantification of ROS positive cells by employing flow cytometric analysis. (**C**) Western blot analysis of HIF-1α and HIF-2α protein levels in H/3.1 and H/SB3 cells. In some experiments H/SB3 cells were pretreated with Rotenone (Rot, 2.5 µM), DPI (1 µM), or Apocynin (APO, 250 µM) at the indicated time. Equal loading was evaluated by re-probed membranes for α-tubulin. BIORAD Quantity One software was used to perform the densitometric analysis (data are expressed as Fold Change relative to the normalized control condition expression). (**D**) Western blot analysis of phosphorylated ERK performed on H/3.1 and H/SB3 cells at 6 h. In some experiments, cells were pretreated with Rotenone (Rot, 2.5 µM) or DPI (1 µM). Equal loading was evaluated by re-probed membranes for total ERK. BIORAD Quantity One software was used to perform the densitometric analysis (data are expressed as Fold Change relative to the normalized control condition expression). (**E**) Western blot analysis of HIF-1α protein levels in H/3.1 and H/SB3 cells treated or not with ERK pharmacological inhibitor PD98059. Equal loading was evaluated by re-probing membranes for α-tubulin. BIORAD Quantity One software was used to perform the densitometric analysis (data are expressed as Fold Change relative to the normalized control condition expression). (**F**) qPCR analysis of HIF-1α transcript levels in control H/3.1 or H/SB3 cells. In the same experiments H/SB3 cells were pretreated with Rotenone (Rot, 2.5 µM) and DPI (1 µM). Data are expressed as means ± SEM of three independent experiments. ** *p* < 0.01 vs. H/3.1 and # *p* < 0.05 vs. related H/SB3 cells.

Although HIF-1α expression may be modulated by several non-hypoxic stimuli (i.e., growth factors, cytokines, hormones like angiotensin II, thrombin) [22], growing evidence indicates that reactive oxygen species (ROS) can mediate HIF-1α transcriptional and translational regulation, specifically through ERK and PI3K/AKT pathways [23,24]. In our experiments, we detected an early and transient increase in intracellular ROS in H/SB3 cells as compared with control H/3.1 cells (Figure 2A,B). ROS generation was almost completely abolished by pretreating H/SB3 cells with Rotenone an inhibitor of complex I of mitochondrial electron chain or by the inhibitor of flavin-dependent enzymes diphenyleneiodonium (DPI) (Figure 2A,B), while it was unaffected by the addition of the pan-NADPH-oxidase inhibitor apocynin (APO) (Figure 2A), suggesting that SB3 elicited ROS release by mitochondria. Accordingly, SB3-dependent up-regulation of HIF-1α and activation of ERK1/2 signaling pathway were prevented by pretreating H/SB3 cells with either Rotenone or DPI (Figure 2C,D) or with pharmacological inhibitor of the ERK pathway (PD98059) (Figure 2E) but unaffected by APO (Figure 2C). Rotenone and DPI also reduced HIF-1α transcript levels (Figure 2F). By contrast, the use of ROS inhibitors was ineffective in reducing HIF-2α protein levels (Figure 2C).

Interestingly, immunohistochemistry analyses performed on liver serial sections from both SB3-TG mice and SB3-positive human HCC specimens (in comparison with specimens from normal liver, Figure S1), showed an intense staining for HIF-1α in the same tissue areas positive for heme-oxygenase-1 (HO-1) (Figure S2A,B), known to be up-regulated by HIF-1α through ROS [25,26]. By contrast, immune-positivity for SB3, HIF-1α, and HO-1 was almost absent in SB3 KO mice. Moreover, the highest levels of HO-1 transcripts were found in the sub-class of patients carrying higher (>than median value) SB3 expression (Figure S2C). Statistical analyses in human specimens also revealed a positive correlation (Spearman r coefficient = 0.3892, *p* < 0.01) between HIF-1α and HO-1 transcript levels (Figure S2D). Furthermore, the highest levels of HIF-1α transcripts were found in the sub-class of patients characterized by higher levels (> than median value) of HO-1 transcripts (Figure S2E). Based on the data obtained in human HCCs, we analyzed HCC nodules from murine specimens obtained from a rodent model of hepatocarcinogenesis based on the administration to mice of the hepato-carcinogen dimethyl-nitrosoamine (DEN) in combination with the choline-deficient L-amino acid refined (CDAA) diet. HIF-1α, HO-1 and SB3 transcript levels were all up-regulated in HCC nodules (Figure S3A–C). Moreover, the highest levels of HO-1 mRNA were found in nodules carrying higher (> than median value) SB3 expression (Figure S3D). Here again, statistical analyses on HCC nodules revealed a positive correlation (Spearman r coefficient = 0.4512, *p* < 0.05) between HIF-1α and HO-1 transcript levels (Figure S3E), with the highest levels of HIF-1α transcripts detected in HCC nodules characterized by higher levels (> than median value) of HO-1 transcripts (Figure S3F).

### 2.3. SerpinB3 Induces HIF-2α Stabilization through Selective NEDDylation

According to previous results, we next investigated whether SB3-induced HIF-2α up-regulation was dependent on post-translational modifications leading to HIF-2α recruitment/stabilization.

**Figure 3:**
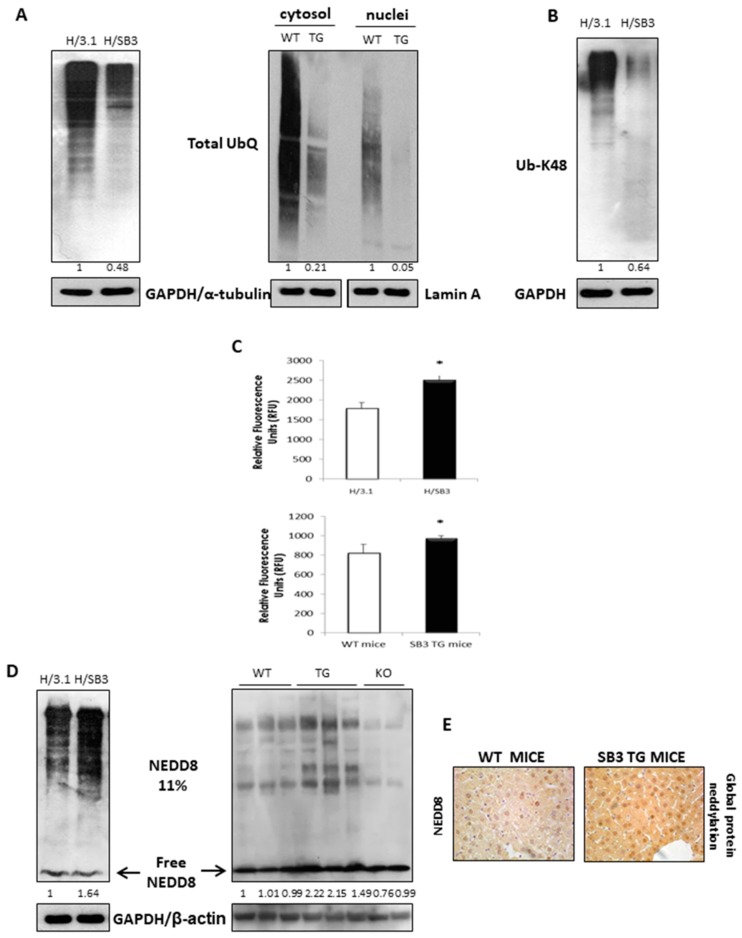
Post-translational modifications: ubiquitination of proteins and 26s proteasome activity versus NEDDylation. (**A**) Western blot analysis of pan-Ubiquitin in H/3.1 or H/SB3 as well as on cytosolic and nuclear fractions obtained from WT and SB3-TG mice. Equal loading was evaluated by re-probing membranes for gliceraldehyde-3-phosphate dehydrogenase (GAPDH) or α-tubulin (for total extract and cytosolic fraction, respectively) or lamin-A (for nuclear fraction). BIORAD Quantity One software was used to perform the densitometric analysis (data are expressed as Fold Change relative to the normalized control condition expression). (**B**) Western blot analysis of K48-linked poly-ubiquitination in H/3.1 or H/SB3 cells. Equal loading was evaluated by re-probing the membrane for GAPDH. BIORAD Quantity One software was used to perform the densitometric analysis (data are expressed as Fold Change relative to the normalized control condition expression). (**C**) 26S proteasome activity obtained from H/3.1 and H/SB3 cells as well as from livers of WT (Wild type mouse-strain C57BL6J) and SB-TG (Transgenic for human SerpinB3-strain C57BL6J) mice. Data are expressed as means of Relative Fluorescence Units (RFU) ± SD from three independent experiments (* *p* < 0.05 vs. control values). (**D**) Western blot analysis of Neural precursor cell Expressed Developmentally Downregulated-8 (NEDD8) on total extracts obtained from control H/3.1 cells and H/SB3 cells as well as on total extract from WT, SB3-TG, and SB3-KO mice showing increased levels of NEDDylated proteins when SB3 was over-expressed. Equal loading was evaluated by re-probing membranes for GAPDH or β-actin. BIORAD Quantity One software was used to perform the densitometric analysis (data are expressed as Fold Change relative to the normalized control condition expression). (**E**) In vivo IHC analysis for NEDD8 (global protein neddylation) on WT mice and SB3-TG mice.

As a first approach, according to literature knowledge on HIF-2α changes leading to ubiquitination and degradation by the 26s proteasome under normoxia [27], we hypothesized that the SB-3 dependent stabilization of HIF-2α was due to a dysfunction of the ubiquitin-proteasome machinery. Analyzing the total ubiquitination status of proteins in either total extracts from H/3.1 and H/SB3 or in cytosolic and nuclear fractions proteins of liver specimens from SB3-TG and WT mice, we found a decrease of total ubiquitinated proteins when SB3 is overexpressed (Figure 3A). In particular, the analysis of poly-ubiquitination on Lys48, a specific tag for addressing proteins to proteasome degradation [28,29], showed a decrease of Lys48-mediated ubiquitination in H/SB3 vs. H/3.1 cells (Figure 3B). Accordingly, we found a significant increase of the proteasome 26s activity in both H/SB3 cells or SB3-TG mice (Figure 3C). These results led us to reasonably exclude the ubiquitin-proteasome system as being involved in HIF-2α post-translational modifications and to consider alternative mechanisms by which HIF-2α may be stabilized in H/SB3 cells.

We then next focused our attention on the involvement of a new class of proteins called Ubiquitin-Like proteins (UBLs) since these molecules are structurally and functionally related to ubiquitin. In particular, the Neural precursor cell Expressed Developmentally Downregulated-8 (NEDD8) conjugation pathway (i.e., NEDDylation) has several analogies with ubiquitination, resulting in the reversible covalent conjugation of a molecule of NEDD8 to a lysine residue of the substrate proteins [30]. However, by competing with ubiquitin, the binding of NEDD8 molecules to target proteins is reported to promote their stabilization. Emerging literature data reported that the NEDDylation process was detected in HCC and associated with its poorest prognosis [31]. Along these lines we next investigated the involvement of a NEDDylation process in SB3-dependent stabilization of HIF-2α. H/SB3 cells and SB3-TG mice showed a significant increase in total NEDDylated proteins (Figure 3D,E), an event not associated to a corresponding increase in monomeric NEDD8 (free NEDD8), whose levels were comparable to those detected in H/3.1 cells and WT littermates (Figure 3D), respectively. Moreover, total NEDDylated proteins were reduced in SB3-KO mice (Figure 3D) further confirming an involvement of SB3 in the NEDDylation process.

**Figure 4:**
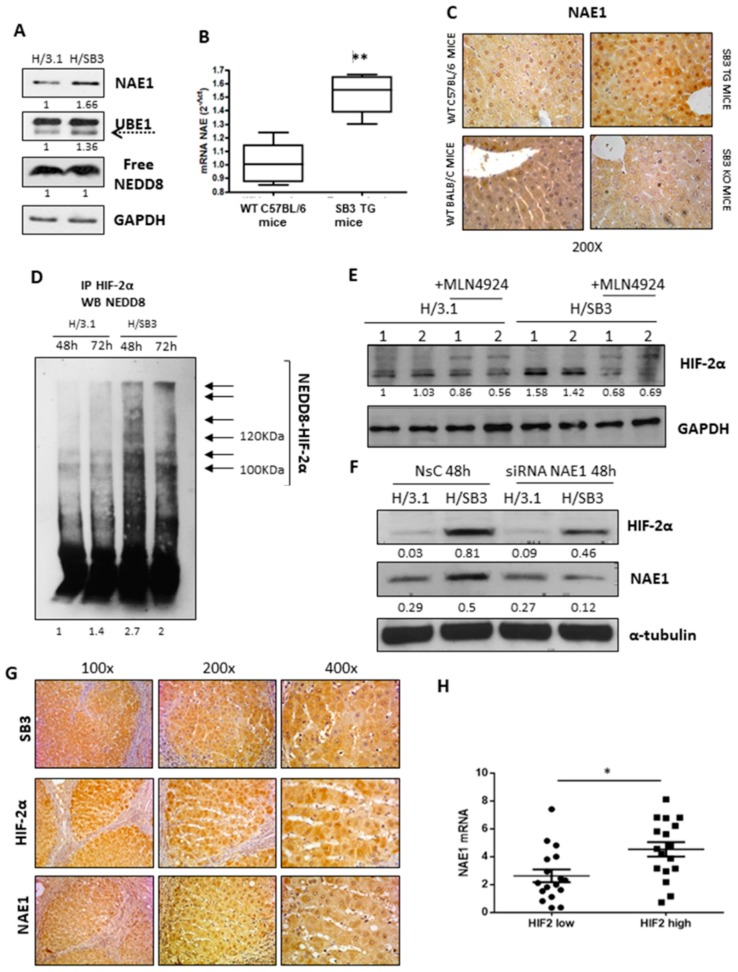
SB3-dependent stabilization of HIF-2α through direct NEDDylation. (**A**) Western blot (WB) analysis of NEDD8-E1 activating enzyme (NAE1) and ubiquitin-activating (E1) enzyme family (UBE-1) in control H/3.1 and H/SB3 cells showing increased levels of both protein in H/SB3. Equal loading was evaluated by re-probing membrane for GAPDH. BIORAD Quantity One software was used to perform the densitometric analysis (data are expressed as Fold Change relative to the normalized control condition expression). (**B**) qPCR analysis of NAE1 transcript levels in WT and SB3-TG mice. Data are expressed as means ± SEM of three independent experiments (** *p* < 0.01 vs. WT littermates). (**C**) IHC analysis of NAE1 performed on WT (mouse-strain C57BL6J), TG (Transgenic for human SerpinB3-strain C57BL6J), BC (Wild type mouse-strain Balb/c), and KO (Knock-out for mouse Serpinb3a-strain Balb/c). Original magnification is indicated. (**D**) Western blot analysis of NEDD8 after immunoprecipitation for HIF-2α in H/3.1 and H/SB3 cells at the indicated time points. BIORAD Quantity One software was used to perform the densitometric analysis (data are expressed as Fold Change relative to the normalized control condition expression). (**E**) WB analysis of HIF-2α obtained from H/3.1 or H/SB3 cells. In some conditions, cells were treated with a specific inhibitor of NAE1 (MLN4924, 3 μM) for 24 h. Equal loading was evaluated by re-probing membranes for GAPDH. BIORAD Quantity One software was used to perform the densitometric analysis (data are expressed as Fold Change relative to the normalized control condition expression). (**F**) WB analysis of HIF-2α and NAE-1 in H/3.1 and H/SB3 cells, transfected with non-silencing control (NsC) siRNA or NAE-1 specific siRNA. Equal loading was evaluated by re-probing membranes for α-tubulin. BIORAD Quantity One software was used to perform the densitometric analysis (data are expressed as Fold Change relative to the normalized control condition expression). (**G**,**H**) Correlation between HIF-2α and NEDDylation in human hepatocellular carcinoma (HCC) specimens. (**G**) IHC analysis for NAE-1, HIF-2α, and SB3 performed on liver sections from HCC patients (grade G2) showing immune-positive staining for NAE1 and HIF-2α in the same areas also positive for SB3. Original magnification is indicated. (**H**) NAE1 and HIF-2α mRNA were analyzed by qPCR in HCC specimens in relation to low expression (HIF-2α low; *n* = 17) or high expression (HIF-2α high; *n* = 17) of HIF-2α mRNA. The y axis represents the relative mRNA amounts of the normalized gene. Central bars represent mean and external bars represent SEM. The analysis was performed with Mann–Whitney test showing significant correlation between HIF-2α and NAE1 (* *p* < 0.05).

The lack of changes in monomeric NEDD8 levels was consistent with the increased protein levels of NEDD8-E1 activating enzyme (NAE1) and of canonical member of the Ubiquitin-activating (E1) enzyme family (UBE-1) [30,32] in H/SB3 (Figure 4A) as well as of NAE1 transcripts in SB3-TG versus WT mice (Figure 4B). In addition, IHC analysis revealed higher nuclear immune-positivity for NAE1 in SB3-TG as compared to WT mice as well as a reduction of cytoplasmic and nuclear staining of NAE1 in KO mice (Figure 4C). Immunoprecipitation using antibody against HIF2α and Western blot analysis using NEDD8 antibody confirm the direct NEDDylation of HIF2α (Figure 4D). In particular, only in H/SB3 cells a number of additional protein bands for NEDD8 were observed at higher apparent molecular weight, suggesting NEDDylation and polymerized/aggregated HIF2α. This is not surprising since NEDDylation of target protein aggregates has been already reported by Maghames et al. [33]. Moreover, HIF-2α protein levels were significantly reduced in H/SB3 cells following pretreatment with MLN4924 (Figure 4E), the first in class inhibitor of NAE1 and NEDD8 pathway [30,34], or after effective silencing of NAE1 (Figure 4F, Figure S4A). No modification was observed on HIF-1α protein levels (Figure S4B). Along these lines, NEDDylation was shown to be aberrant in liver biopsies of HCC patients in comparison with healthy controls [31,35]. In these patients we also observed a strong positive correlation between the levels of LKB1 and NEDD8 [31]. Consistent with literature data, IHC performed on HCC specimens showed HIF-2α immune-positivity in the same areas also positive for NAE1 and SB3 (Figure 4G), with statistical analyses revealing that the highest levels of NAE1 mRNA were detected in the subclass of HCC patients with highest (> median value) levels of HIF-2α transcripts (Figure 4H).

**Figure 5:**
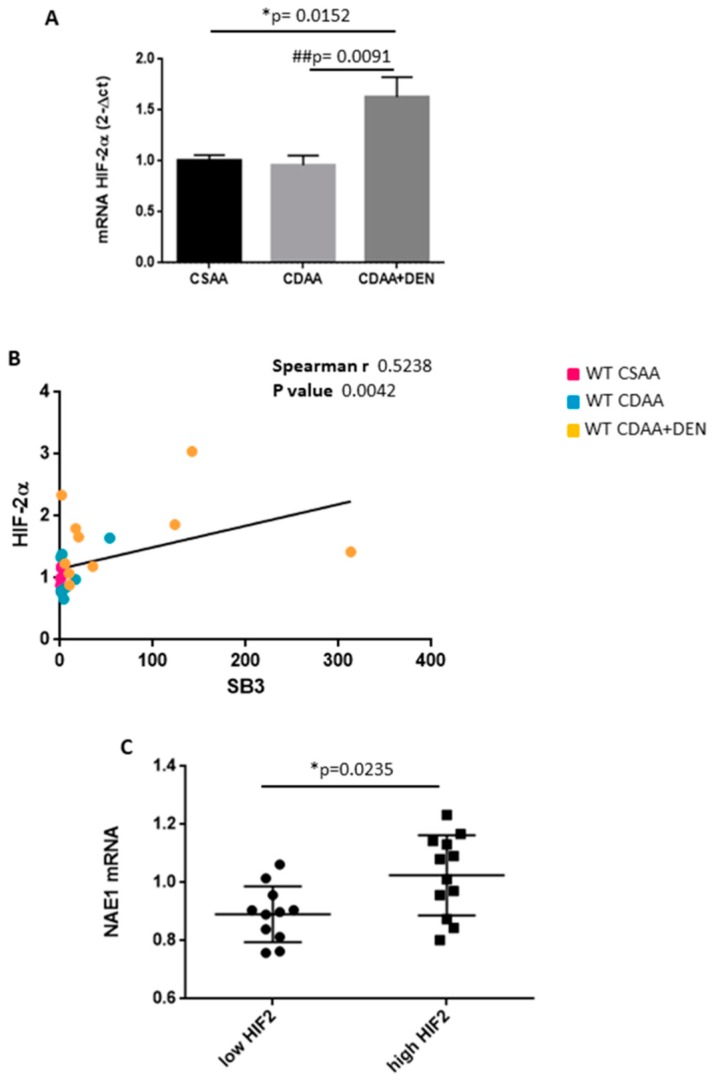
In vivo analysis in HCC nodules obtained from mice undergoing diethyl-nitrosamine (DEN) administration/choline-deficient L-amino acid refined diet (DEN/CDAA) carcinogenic protocol. (**A**) HIF-2α mRNA were analyzed by qPCR. Data are expressed as means ± SEM (* *p* < 0.05 vs. CSAA, ## *p* < 0.01 vs. CDAA). (**B**) HIF-2α and SB3 mRNA were analyzed by qPCR. Statistical analysis was performed using Spearman correlation showing a positive correlation between SB3 and HIF-2α. (**C**) NAE1 and HIF-2α transcripts were analyzed in relation to low expression (HIF-2α low; *n* = 17) or high expression (HIF-2α high; *n* = 17) of HIF-2α mRNA. The y axis represents the relative mRNA amounts of the normalized gene. Central bars represent mean and external bars represent SEM. The analysis was performed with Mann–Whitney test showing a significant correlation between HIF-2α and NAE1 (* *p* < 0.05).

Finally, analyzing HCC nodules from rodent model of hepatocarcinogenesis based on the administration to mice of the hepato-carcinogen dimethyl-nitrosoamine (DEN) in combination with the CDAA diet, we observed that (i) HIF-2α transcript levels were up-regulated in HCC nodules (Figure 5A); (ii) there was a positive correlation (Spearman r coefficient = 0.5238, *p* < 0.01) between HIF-2α and SB3 (Figure 5B); (iii) the highest levels of NAE-1 transcripts were found in the sub-class of nodules carrying higher (> than median value) HIF-2α expression (Figure 5C).

### 2.4. SerpinB3 Up-Regulated HIF-α Subunits Are Biologically Active

**Figure 6:**
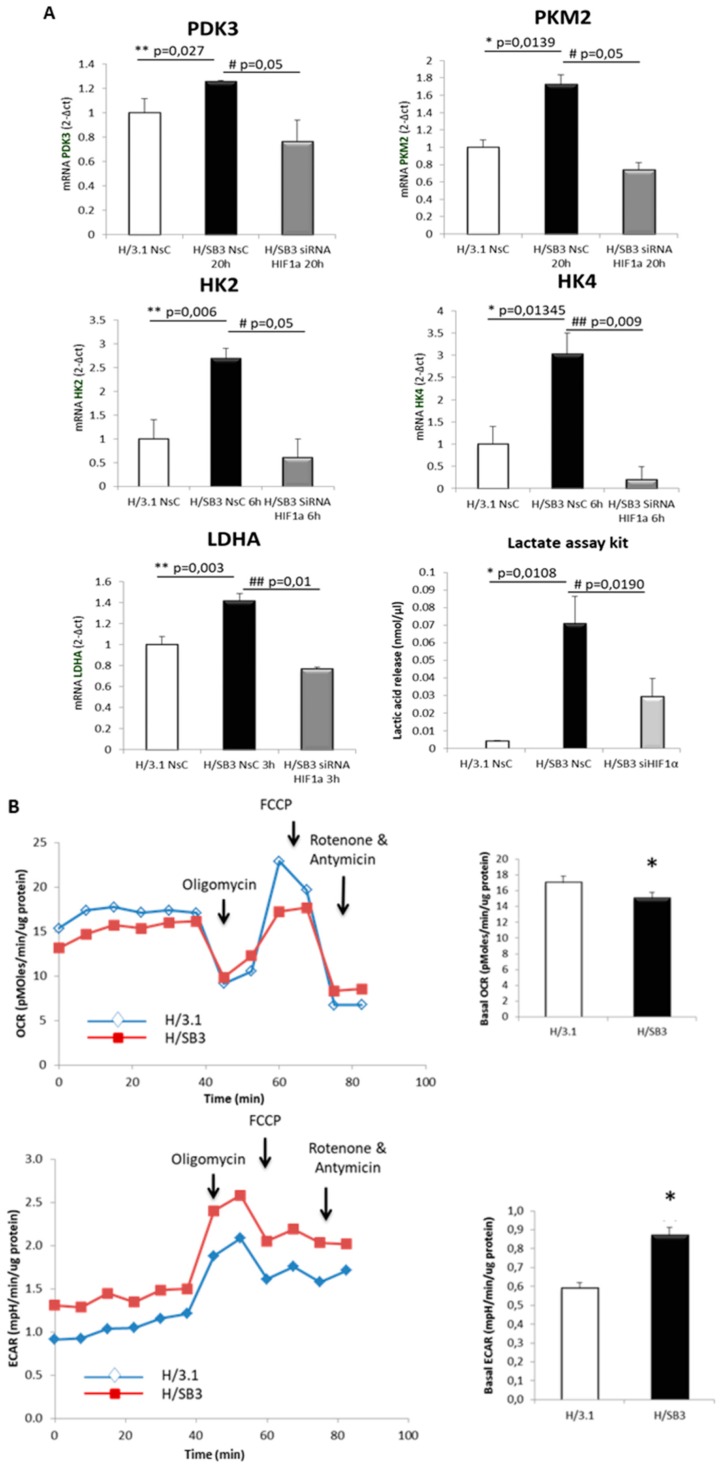

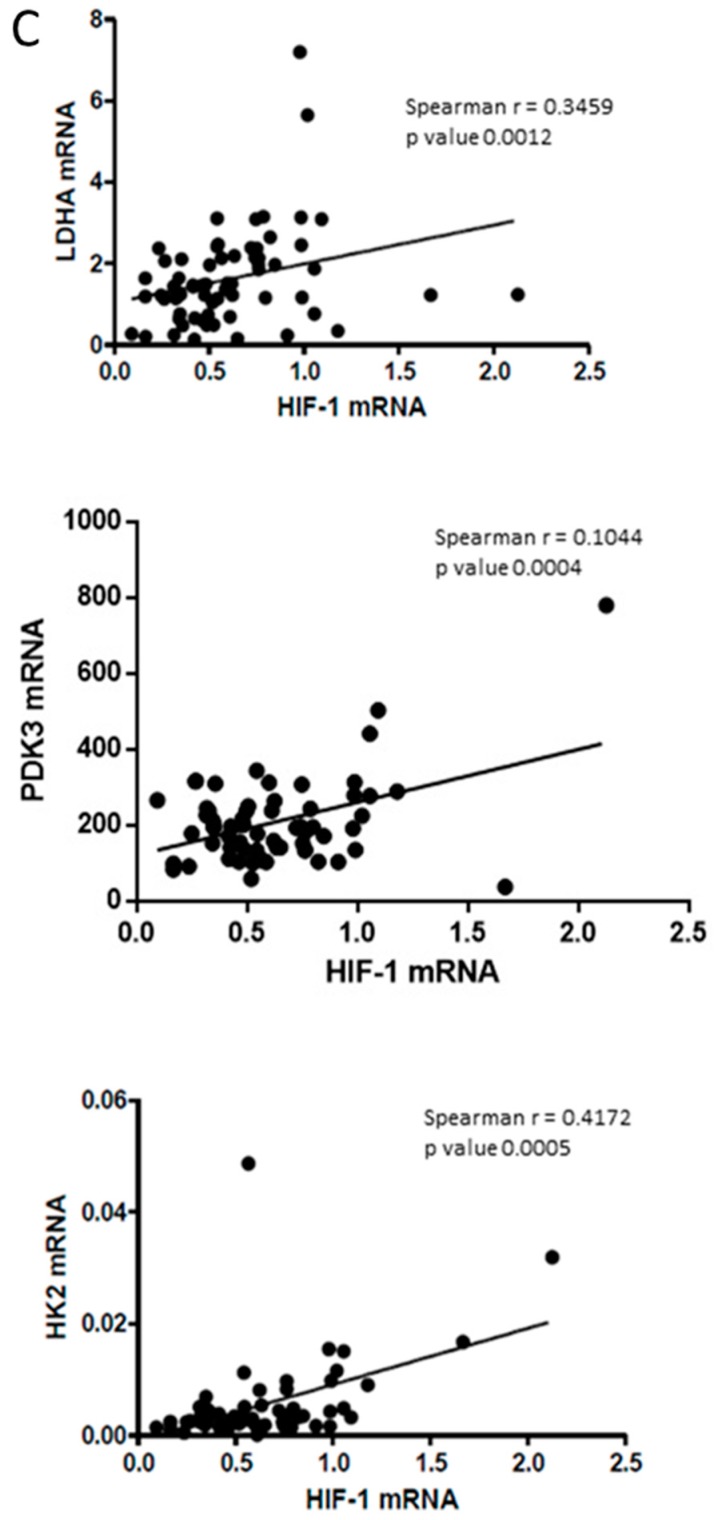
HIF-1α-dependent switch to glycolytic phenotype of H/SB3. (**A**) qPCR analysis of HIF-1α-target genes (*PDK3*, *PKM2*, *HK2*, *HK4*, and *LDHA*) and lactate release in H/3.1 and H/SB3 transfected with non-silencing siRNA (NsC) or with specific siRNA for HIF-1α (siHIF-1α). Data are expressed as means ± SEM of three independent experiments (* *p* < 0.05 vs. H/3.1, ** *p* < 0.01 vs. H/3.1, # *p* < 0.05 vs. related control condition, ## *p* < 0.01 vs. related control condition). (**B**) Seahorse Bioscence XF Analyzers of OCR (Oxygen Consumption Rate) and ECAR (Extracellular Acidification Rate) parameters in H/3.1 or H/SB3 cells. * *p* < 0.05 vs. H/3.1. Data are expressed as means ± SD of three independent experiments. (**C**) HIF-1α, LDHA, PDK3, and HK2 mRNA were analyzed by qPCR that was performed in liver specimens from 67 patients carrying HCC. The use of nonparametric Spearman test showed the presence of a positive correlation between HIF-1α and related target genes *LDHA*, *PDK3*, *HK2*; Spearman r coefficient and *p* value were indicated.

We next performed experiments to verify whether SB3-dependent up-regulation of HIF-α subunits resulted in increased expression of related target genes. According to literature, HIF-1α is mainly involved in the regulation of glycolytic gene expression during O_2_ deprivation, whereas HIF-2α overexpression correlates with poor patient outcome in several human cancers and seems to be associated with tumor growth and progression [36]. In our experiments we found that SB-3-dependent HIF-1α up-regulation led to increased expression of genes involved in glycolysis, including Pyruvate Dehydrogenase Kinase isozyme-3 (PDK3), Pyruvate Kinase Muscle (PKM2), Hexokinase 2 (HK2), Hexokinase-4 (HK4), and Lactate Dehydrogenase A (LDHA), resulting in increased lactate release in culture medium of H/SB3 (Figure 6A). Moreover, following specific HIF-1α silencing (Figure S6A), we observed a significant reduction of HIF-1α target genes as well as of lactate release in culture medium (Figure 6A). Accordingly, specific silencing of HIF-2α was ineffective on transcript levels of LDHA, used as most representative glycolytic gene target (Figure S8A).

Moreover, by employing Seahorse analysis to evaluate the real-time measurement of cellular bioenergetics, we observed an effective and significant reduction of basal oxygen consumption rate (OCR) as well as an increase in extracellular acidification rate (ECAR) in normoxic H/SB3 versus H/3.1 cells (Figure 6B), events that were reverted by using a specific siRNA for HIF-1α (Figure S6A,B).

By performing qPCR on HCC specimens obtained from a cohort of 67 well characterized patients employed in a previous study [37], we analyzed transcript levels of both HIF-1α and glycolytic genes. Statistical analysis extended to all available specimens led us to highlight the existence of a significant positive correlation between HIF-1α and LDHA, PDK3 and HK2 transcript levels (Figure 6C), confirming that also in vivo HIF-1α may trigger the glycolytic phenotype switch.

**Figure 7:**
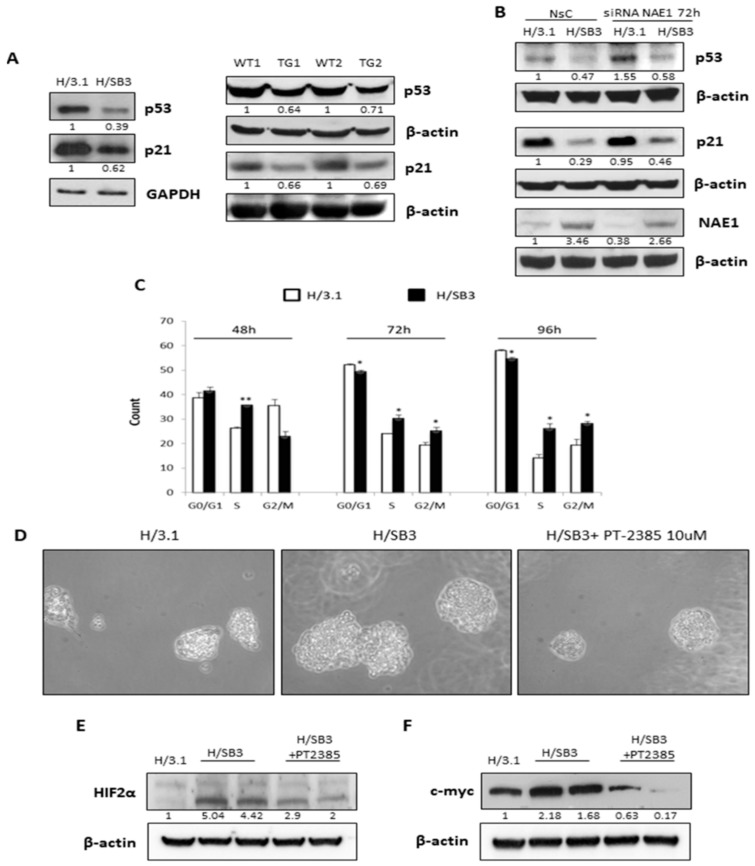

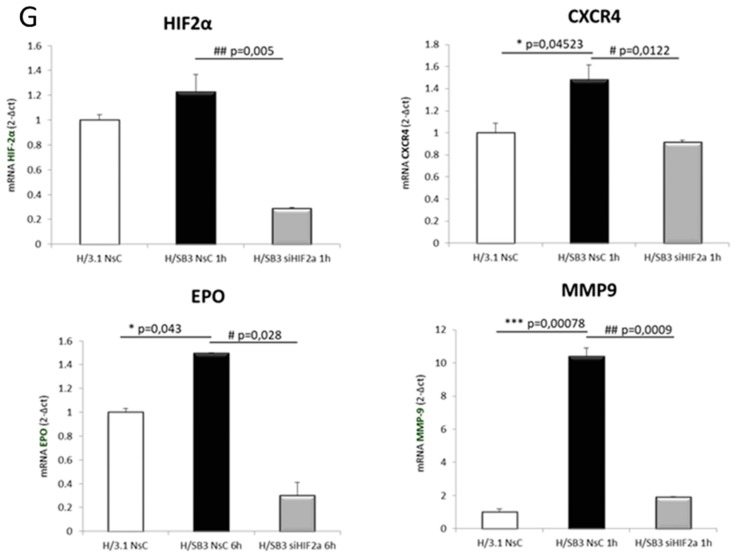
HIF-2α stabilized by SB3 is involved in cell cycle regulation and potentially mediates cancer progression. (**A**) Western blot analysis of p53 and p21 performed on H/3.1 and H/SB3 cells as well as in WT and SB3-TG mice. Equal loading was evaluated by re-probing membranes with GAPDH and β-actin. BIORAD Quantity One software was used to perform the densitometric analysis (data are expressed as Fold Change relative to the normalized control condition expression). (**B**) Western blot analysis of p53, p21, and NAE-1 performed on H/3.1 and H/SB3 cells transfected with non-silencing siRNA (NsC) or with specific siRNA for NAE1. Equal loading was evaluated by re-probing membranes for β-actin. BIORAD Quantity One software was used to perform the densitometric analysis (data are expressed as Fold Change relative to the normalized control condition expression). (**C**) Bar graph charts showing the relative quantity of G0/G1, S, and G2/M ratio in H/SB3 cells compared to the H/3.1 control cells as mean ± SD, resulted from cell cycle analysis by flow cytometry with FCS Express 4 Flow Research Edition software. This experiment was repeated three separate times, and similar results were obtained. (* *p* < 0.05 or ** *p* < 0.01 vs. H/3.1 cells). (**D**) Soft agar colony formation assay on H/3.1 and H/SB3 cells. In some experiments H/SB3 cells were treated with HIF-2α specific inhibitor (PT-2385, 10 µM). (**E**,**F**) WB analysis of HIF-2α (E) and c-myc (F) protein levels performed on H/3.1 and H/SB3 cells treated or not with HIF-2α specific inhibitor (PT-2385, 10 µM). Equal loading was evaluated by re-probing membranes for β-actin. BIORAD Quantity One software was used to perform the densitometric analysis (data are expressed as Fold Change relative to the normalized control condition expression). (**G**) qPCR analysis of HIF-2α and related target genes (MMP-9, CXCR4, and EPO) in H/3.1 or H/SB3 cells transfected with non-silencing siRNA (NsC) or with specific siRNA for HIF-2α (siHIF-2α) at the indicated time points. Data are expressed as means ± SEM of three independent experiments (* *p* < 0.05 and *** *p* < 0.001 vs. H/3.1; # *p* < 0.05 and ## *p* < 0.001 vs. related control condition).

On the other hand, SB3-dependent HIF-2α stabilization resulted in a reduction of p53 and p21 protein levels in both H/SB3 normoxic cells and SB3-TG mice (Figure 7A). Although the in vivo reported a decrease in p53 and p21 protein levels in SB3-TG mice may result from more than one mechanism, in vitro experiments showed that NAE1 specific silencing, which re-established the normal HIF-2α normoxic degradation, partially abolished the HIF-2α-dependent reduction of p53 and p21 proteins observed in H/SB3 cells (Figure 7B). In addition H/SB3 cells showed up-regulation of c-myc protein levels and reduction of p21 and p53 protein levels (Figure S7A,B), events reverted by HIF-2α silencing (Figure S7C), thus supporting the involvement of HIF-2α in the regulation of cell cycle regulators (p53 and p21) and tumor progression (c-myc). Accordingly, HIF-1α silencing in H/SB3 cells was ineffective on transcript levels of Myc, p53, and p21 (Figure S8B). Such a SB-3-related and HIF-2α-dependent regulation of p53, p21, and c-Myc resulted in a shift of cell cycle towards S phase (increase in S phase ratio up to 96 h), then indicating that H/SB3 cells are more proliferative than control H/3.1 cells (Figure 7C), with cells in G2/M phase increasing later on at 72 and 96 h. In addition, performing soft agar colony formation assay [9], the most stringent tests for malignant transformation in cells which measures the ability of cells to proliferate in semi-solid matrices, we demonstrated that the increase in colony formation and dimension of H/SB3 was prevented by using the HIF-2α specific inhibitor (PT-2385 10 µM [38,39], Figure 7D) that efficiently down-regulated in H/SB3 cells HIF-2α and c-myc protein levels (Figure 7E,F).

Moreover, HIF-2α target genes involved in tumor progression such as metalloproteinase 9 (*MMP-9*), C-X-C chemokine receptor type 4 (*CXCR4*, receptor of the chemokine SDF-1), and the more generic target gene erythropoietin (*EPO*) are up-regulated in H/SB3 cells and decrease by using HIF-2α specific siRNA (Figure 7G).

### 2.5. SerpinB3 Was Able to Modulate Angiogenesis

**Figure 8:**
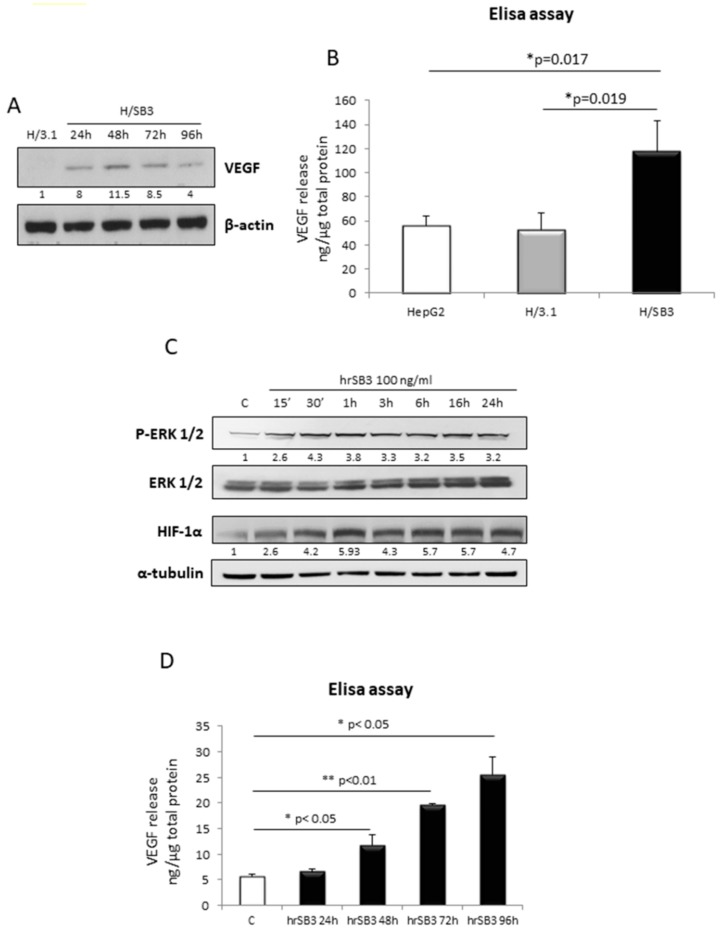

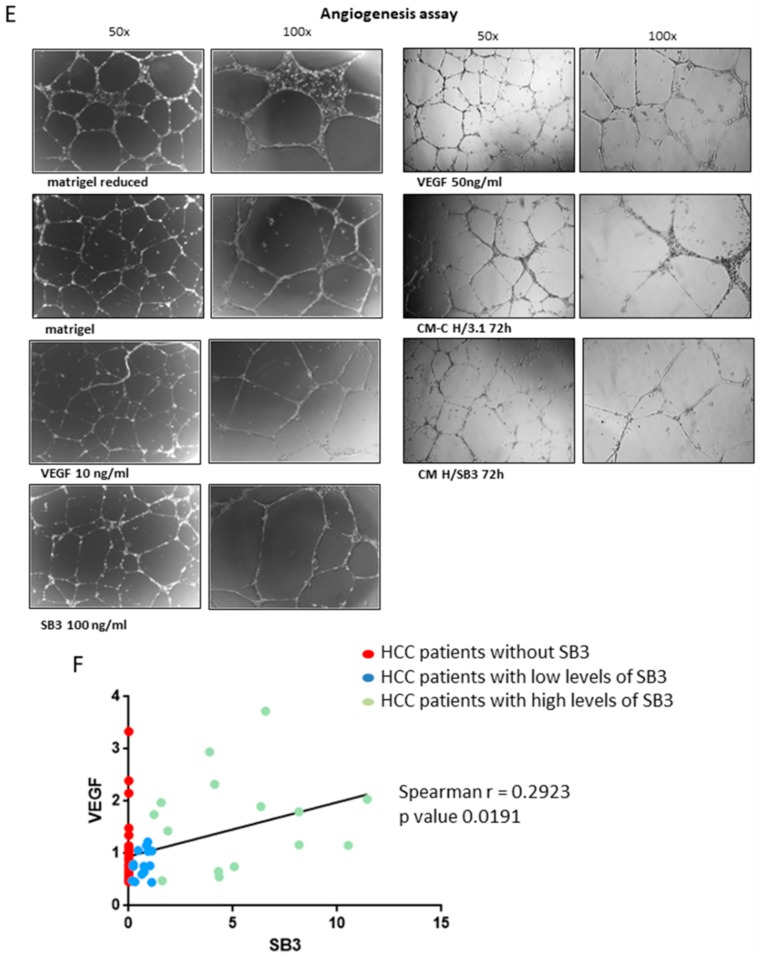
SB3 induces angiogenesis. (**A**) WB analysis of VEGF performed on H/3.1 and H/SB3 cells at indicated time points (time-course analysis started after 20 h from cell seeding). BIORAD Quantity One software was used to perform the densitometric analysis (data are expressed as Fold Change relative to the normalized control condition expression). (**B**) VEGF ELISA assay on H/3.1 and H/SB3 at 72 h. Data are expressed as means ± S.D of three independent experiments (* *p* < 0.05 vs. H/3.1). (**C**) WB analysis of pERK1/2 and HIF1α performed on HepG2 naïve cells treated with human recombinant SB3 (hrSB3, 100 ng/mL) at indicated time points. Equal loading was evaluated by re-probing membranes with total ERK or β-actin, respectively. BIORAD Quantity One software was used to perform the densitometric analysis (data are expressed as Fold Change relative to the normalized control condition expression). (**D**) VEGF ELISA assay on HepG2 naïve treated with hrSB3 at indicated time points. Data are expressed as means ± S.D of three independent experiments (* *p* < 0.05 or ** *p* < 0.01 vs. HepG2 control cells). (**E**) HUVEC tubulogenesis assays were performed in solidified basement membrane matrix (Matrigel; BD Biosciences). HUVECs were seeded and treated or not with the hrSB3 (100 ng/mL) as well as with conditioned medium collected from H/3.1 control cells (CM-C H/3.1) or H/SB3 cells (CM-H/SB3) after 72 h (VEGF-A 10 ng/mL or 50 ng/mL was used as positive control). After 14 h, tubular structures were microscopically examined and photographed (Leica, Wetzlar, Germany). (**F**) SB3 and VEGF mRNA were analyzed by qPCR that was performed in liver specimens from 67 patients carrying HCC. The use of non-parametric Spearman test showed the presence of a positive correlation between VEGF and SB3 (* *p* < 0.05).

To integrate the overview of the biological effects of SB3 on HCC cells and tumor microenvironment, we evaluated the ability of SB3 to modulate angiogenesis. H/SB3 cells, as compared to H/3.1 cells, showed increased VEGF intracellular protein levels (Figure 8A), as well as an increased release of VEGF in the extracellular medium (Figure 8B).

Accordingly, HepG2 naïve cells responded to the addition of human recombinant SB3 (hrSB3 100 ng/mL) led to the phosphorylation and activation of ERK1/2, to HIF1α induction (Figure 8C) resulting in a time-dependent release of VEGF in culture medium (Figure 8D).

Moreover, by performing tubulogenesis assay on HUVEC cells treated with either hrSB3 or conditioned medium from H/SB3 cells (CM-H/SB3), we found that SB3 was able to induce tubes formation (Figure 8E, human recombinant VEGF used as positive control), indicating that SB3 can act as a paracrine mediator able to favor angiogenesis in the tumor microenvironment.

Finally, we performed an additional qPCR analysis for SB3 and VEGF transcript levels in liver specimens from 67 patients carrying HCC. The use of non-parametric Spearman test showed the presence of a positive and significant correlation (Spearman r coefficient = 0.2923, *p* < 0.05) between these transcripts, then supporting the hypothesis of a strict relationship between angiogenesis and SB3 (Figure 8F).

## 3. Discussion

The present study provides evidence supporting the novel concept that SB3, in the contest of tumor microenvironment, can operate as a paracrine mediator able to differentially up-regulate HIF-1α (i.e., through redox-dependent increased transcription) and HIF-2α (through selective NEDDylation) protein levels in a hypoxia-independent manner.

The early and transient up-regulation of HIF-1α protein levels in H/SB3 cells was correlated to SB3-dependent increased intracellular ROS levels (likely released by mitochondria). Up-regulation of HIF-1α by ROS has been extensively described to occur early in cells exposed to hypoxia [24,25,26,40], but a growing body of evidence also suggests that ROS and related mechanisms and pathways can mediate HIF-1α up-regulation even under normoxia [23] through different signaling pathways, including ERK and PI3K/Akt pathways. According to literature data, results obtained in this study showed that transcriptional regulation of HIF-1α is more likely related to ROS-dependent activation of the ERK pathway.

Even more relevant, this study provides the novel message that SB3 can trigger selective NEDDylation of HIF-2α as a mechanism able to stabilize and increase its protein levels. It is well known that HIF-α subunits are rapidly and continuously degraded in normoxia as a consequence of hydroxylation on proline residues (Pro402-564 for HIF-1α and Pro405-531 for HIF-2α) in their ODD (Oxygen-dependent Degradation Domain) domains [41]. This usually leads to α-subunit ubiquitination by Von Hippel Lindau (VHL)-ubiquitin-E3 ligase and consequent proteasomal degradation. Our data from in vivo and in vitro experimental models indicate that the prevention of HIF-2α degradation due to post-translational modification is not associated to ubiquitin-proteasome system dysfunction. In particular, poly-ubiquitination on K48-lysine residues, which is an event targeting protein for proteasome-mediated degradation [28,29], was decreased in H/SB3 cells, as a consequence of the increased activity of proteasome machinery.

Accordingly, we next considered other post-translational modifications of cellular proteins, by focusing our attention to the involvement of NEDD8 and NEDDylation process in the stabilization of proteins with essential regulatory roles [30]. Concerning NEDD8-related target proteins, the substrates were divided into cullins and non-cullins, including p53, MDM2, pVHL, HIF-1α/HIF-2α, HuR, and more [42,43]. Cullins are a major family of proteins that are subunits of the E3 ligase machinery, which brings together E2 ubiquitin-conjugating enzymes and substrates that have been targeted for degradation. In relation with HIFs, Ivan and colleagues [44] reported the involvement of Cullin 2 (Cul2) in the ubiquitin-ligase complex that binds pVHL, leading to HIF-1α poly-ubiquitination and its consequent O_2_-dependent degradation. Controversial data have been reported about the role of NEDDylation in the regulation of HIF-1α protein levels: Curtis and colleagues [45] reported that NEDDylation of Cul2 facilitates HIF-1α poly-ubiquitination and proteasome degradation; indeed the inhibition of Cullin-2 NEDDylation by using the NAE1 pharmacological inhibitor that resulted in HIF-1α stabilization, activation of HIF promoter, and up-regulation of HIF-target genes in human epithelial intestinal cells and HeLa cells. By contrast, a study from Russel and Ohh [46] reported that in different cancer cell lines direct NEDDylation of pVHL may negatively regulate HIF-1α ubiquitination leading to its stabilization. Alternatively, in another study by Ryu J-H and colleagues [47], the authors reported that HIF-1α expression and activity were inhibited by knocking down NAE1-mediated NEDD8 conjugation but enhanced by ectopically expressing NEDD8. Moreover, in this study, HIF-1α and HIF-2α were found to be covalently modified by NEDD8 [47]. However, these results were obtained using HEK293 overexpressing HIF-1α, HIF-2α, and NEDD8 and maintained under normoxic condition or in NEDD8-overxpressing cells incubated in hypoxic conditions. Although in our conditions SB3-dependent HIF-1α stabilization under normoxia was clearly ROS-dependent, HIF-1α protein levels were unchanged following NAE1 silencing (Figure S4B), then reasonably excluding a role for SB3-related NEDDylation in HIF-1α stabilization.

On the other hand, our findings demonstrate the involvement of NEDD8 and NEDDylation process in the SB3-dependent and hypoxia-independent stabilization of HIF-2α. Immuno-histochemical and Western blot analyses on SB3-TG mice or H/SB3 cells revealed increased levels of NEDDylated proteins. This event was not associated to up-regulation of monomeric NEDD8, but rather to a SB3-dependent over-expression of the NEDD8-activating enzyme 1 (NAE1). The analysis of HCC specimens as well as of nodules obtained from mice undergoing DEN/CDAA showed a positive and significant correlation between NAE1 and HIF-2α transcript levels was detected, a novel finding which is consistent with data reporting a significant increase in global NEDDylation in human HCC that, in turn, is associated with poorest prognosis [31]. Accordingly, the inhibition of NAE1 by using its specific pharmacological inhibitor MLN4924 as well as NAE1 silencing by employing an efficient and selective siRNA, led to the selective reduction of HIF-2α protein levels.

In our experimental models, the ability of SB3 to up-regulate and stabilize HIF-1α in a redox-sensitive manner as well as to directly stabilize HIF-2α through NEDDylation resulted in their nuclear translocation (Figure S5) and in selective up-regulation of specific target genes. HIF-1α and HIF-2α are known to exhibit common properties, including a partial sequence identity, the interaction with ARNT and the binding to HRE sequences to induce the expression of common target genes like *GLUT1*, *VEGF-A*, and *ADM-1*. However, literature data also indicate that they can differ significantly for other aspects [36], specifically in their transactivation domains, implying they also have unique target genes [27]. In particular, HIF-1α exclusively regulates glycolytic gene expression, whereas HIF-2α has been shown to promote tumor growth in a renal carcinoma xenograft model [48,49] and invasiveness (through up-regulation of MMP-2, MMP-9, and PAI-1). In the present study we demonstrated that H/SB3 cells display up-regulation of HIF-1α target genes involved in anaerobic glycolysis (*PDK3*, *PKM2*, *HK2*, *HK4*, and *LDHA*) followed by a reduction of OCR, an increase in ECAR parameters as well as an increased release of lactic acid, suggesting that SB-3-dependent aerobic stabilization of HIF-1α could potentially drive glycolysis in tumors without dependence on a hypoxic environment. On the other hand, referring to the partially antithetic activity of HIF-1α and HIF-2α, it has been reported that HIF-1α can lead to cell cycle arrest in hypoxic cells counteracting c-Myc activity [50]. By contrast, cells over-expressing HIF-2α exhibit enhanced c-Myc activity and a more rapid entry in S-phase [36]. Accordingly, here we produced evidence of up-regulation of c-myc protein levels and reduction of p21 and p53 protein levels in H/SB3 cells, events reverted by HIF-2α silencing. This SB-3-related and HIF-2α-dependent regulation of p53, p21, and c-Myc resulted in a shift of cell cycle towards S phase (increase in S phase ratio), that may justify the more proliferative phenotype of H/SB3 cells. These results are consistent with data obtained from soft-agar colony formation assay that demonstrated an HIF-2α-dependent increase in colony formation and dimension on soft-agar of H/SB3 compared to control H/3.1 cells. Moreover, SB3-dependent NEDDylation/stabilization of HIF-2α in H/SB3 normoxic cells resulted in an over-expression of HIF-2α target genes involved in invasiveness and metastasis (MMP-9 and CXCR-4).

## 4. Materials and Methods

### 4.1. Materials

Enhanced chemiluminescence (ECL) reagents, nitrocellulose membranes (Hybond-C extra), and secondary Cy3-conjugated antibodies were from Amersham Pharmacia Biotech Inc. (Piscataway, NJ, USA). The following antibodies were used: anti-HIF-1α (NB100-479) and anti-HIF-2α (NB100-122) from Novus Biologicals; anti SB3 from Xeptagen; anti-total UbQ (sc-8017), anti-LaminA (sc-20680), anti-p53 (sc-6243), anti-p21 (sc-817), anti-gliceraldehyde-3-phosphate dehydrogenase (GAPDH) (sc-20357), and anti-ERK (sc-94) from Santa Cruz Biotechnology; anti-NEDD8 (ab81264) from Abcam; anti-NAE-1 (AP13067c) from Abgent; anti-UBE-1 (BML-PW8395-00251) from ENZO Life Sciences; anti-HO-1 (MA1-112) from Invitrogen; anti-Ub-48 (05-137) from Millipore; anti-P-ERK (#4696) from Cell Signaling Technology; anti-α-tubulin (T9026) and anti-β-actin (A5441) from Sigma Aldrich. DMOG (Dimethyloxalylglycine) and all other reagents of analytical grade were from Sigma Aldrich. HiPerfect Transfection Reagent was from Qiagen. Lactate Colorimetric/Fluorometric assay kit was from Biovision Incorporation.

### 4.2. Methods

#### 4.2.1. Cell Lines and Culture Conditions

HepG2 cells (from ATCC, USA) were stably transfected with the human SB3 cDNA to over-express SB3 (H/SB3) or with the empty vector (pCDNA3.1) alone (H/3.1) as previously described [9]. H/SB3 cells, maintained in Dulbecco’s modified Eagle’s medium as previously described [9] were seeded in normoxic conditions to obtain the desired sub-confluence level (65%–70%) and after 20 h from plating time course analysis was performed from 1 h up to 48 h. 

#### 4.2.2. Mouse Models

The study was carried out on 12–14 week old C57BL/6 mice made transgenic for human SerpinB3, selectively expressing SerpinB3 in hepatocytes (*n* = 6) (with the initial colony kindly provided by Prof. Cassani, Tecnogen, Caserta, Italy) [12], that was already described and employed in previous studies [12,51]. Wild type C57BL/6 mice of similar age (*n* = 6) were used as controls.

Serpinb3a knockout mice (kindly provided by Dr. Gary Silverman and Dr. Cliff J. Luke, University of Pittsburg, Children’s Hospital, Pittsburg, PA) were used (*n* = 5) as an additional negative control murine model. All animals were kept under specific pathogen-free conditions and maintained with free access to pellet food and water at the Animal Care Facility of the Experimental Surgery Division of the University of Padua. Animals experiments and protocols, approved by the local Ethical committee and by the Italian Ministry of Health (the ethical code is N.61319), complied with national ethical guidelines for animal experimentation and were conducted in accordance with the Principles of laboratory animal care (NIH publication no. 85–23, revised 1985; http://grants1.nih.gov/grants/olaw/references/phspol.htm).

In dedicated experiments wild type C57BL/6 mice were submitted to (a) an established liver carcinogenic protocol involving a single administration of diethylnitrosamine (DEN, 25 mg/kg bw, i.p.) at the age of 2 weeks to mice that were fed then from the age of 6 weeks on a choline-deficient L-aminoacid-defined (CDDA) diet (Laboratorio Dottori Piccioni, Gessate, Italy) for additional 26 weeks [52] (b) a CDAA diet only or (c) a choline-sufficient L-aminoacid-defined (CSSA) diet (Laboratorio Dottori Piccioni, Gessate, Italy) for 26 weeks starting from the age of 6 weeks. Mice were kept under specific pathogen-free conditions and maintained with free access to pellet food and water. Liver samples were obtained and immediately used/processed for morphological or molecular biology analyses or frozen and thereafter maintained at −80 °C for further analysis. These experiments complied with EU and national ethical guidelines for animal experimentation and experimental protocols were approved by the Animal Ethic Commitee of University of Oriental Piedmont, Novara, Italy and Italian Ministry of Health. 

#### 4.2.3. Patients and Samples

Human HCC specimens were obtained, under written informed consent, at the time of surgery in 67 patients with underlying cirrhosis of different etiology. Clinicopathologic and molecular characteristics as well as follow-up procedures applied to these patients were described in a previous study [37]. All the patients underwent surgical resection as first-line therapy without pre-operative anticancer treatment and distant metastases. Tumor samples were either formalin fixed and paraffin embedded or immediately frozen at −80 °C for transcript analysis. The use of human material conforms to the ethical guidelines of the 1975 Declaration of Helsinki and all experimental protocols were approved by the University of Padua Ethical Committee. Informed consent was obtained from all participants included in this study according to the committee regulations.

#### 4.2.4. Quantitative Real-Time PCR (qPCR)

RNA extraction, complementary DNA synthesis, quantitative real-time PCR (qPCR) reactions were performed as previously described [13,37]. The amplification mix was prepared using Roche LightCycler FastStart DNA MasterPLUS SYBR Green I kit following manufacturer’s instructions and real-time PCR was performed using LightCycler instrument. Oligonucleotide sequence of primers used for RealTime qPCR were reported in Table 1. GAPDH was used as internal reference and co-amplified with target samples using identical qPCR conditions. Samples were run in triplicate and mRNA expression was generated for each sample.

#### 4.2.5. NAE1 and HIFs Silencing by Small RNA Interference

RNA interference experiments to knockdown NEDD8 activating enzyme 1 (NAE1), HIF-2α, and HIF-1α expression in HepG2 cells transfected with empty vector or over-expressing SB3 were performed using siRNA duplex and HiPerfect Transfection reagent (Qiagen Italia, Milano, Italy) according to manufacturer’s instructions up to 72 h, as previously described [13,18]. The following target sequences were used:

NAE1: 5′-AAGTAGTGTGTTAATGATTGA-3′
HIF-2α: 5′-CCCGGATAGACTTATTGCCAA-3′
HIF-1α: 5′-AGGAAGAACTATGAACATAAA-3′

#### 4.2.6. Western Blot Analysis

Total cell lysates or nuclear vs. cytosolic extracts were subjected to sodium dodecyl sulfate-polyacrylamide gel-electrophoresis on 20%, 12%, 10%, or 7.5% acrylamide gels as previously described [13,18]. Sample loading was evaluated by reblotting the same membrane with the un-phosphorylated form of protein or with β-actin, α-tubulin, or GAPDH antibody. In some experiments, protein levels in culture medium obtained from H/3.1 or H/SB3 cells were evaluated by an immunoprecipitation procedure, as described previously [53].

#### 4.2.7. Immunohistochemistry Analysis

Paraffin liver sections derived from wild-type mice, SB3 transgenic mice or Sb3a knockout mice as well as human HCC specimens, were immune-stained as previously reported [13,54] by employing 2 μM thick paraffin sections. Immune staining was performed by employing monoclonal or polyclonal antibodies raised against HIF-1α (dilution 1:100), HIF-2α (dilution 1:200), NEDD8 (dilution 1:200), NAE-1 (dilution 1:300), HO-1 (dilution 1:300), as well as polyclonal SB3 antibody (dilution 1:500, for mice specimens) or monoclonal SB3 antibody (dilution 1:50, for human HCC specimens). Endogenous peroxidase was blocked by 3% hydrogen peroxide and antigen retrieval was obtained by microwave; immune positivity was revealed by means of EnVision, HRP-labeled System (DAKO) using 3′-diaminobenzidine as substrate.

#### 4.2.8. Detection of Intracellular Generation of ROS

Detection of ROS generation in cultured cells was performed by using the semi-quantitative 2’,7’-dichlorodihydrofluorescein diacetate (DCFH-DA) technique as previously described [13,18]. H/3.1 or H/SB3 cells (10^5^ cells/well) were seeded in 12-well culture plates and, in some experiments, pretreated with either Apocynin (APO, 250 µM), Rotenone (Rot, 2.5 µM), or DPI (1 µM). Intracellular generation of ROS was detected using 2′,7′-dichlorodihydrofluorescein diacetate which, in the presence of ROS, is converted to a fluorescent derivative. Fluorescent cells were then analyzed using a Leica fluorescence microscope (DMI 4000 B model). Alternatively, detection of ROS generation was evaluated by combining DCFH-DA technique and flow cytometric analysis. Cells were seeded in P35 dishes (5 × 10^5^ cells/dish) and exposed to hrSB3 alone or hrSB3 plus Rotenone 2.5 µM or DPI 1 µM before addition of DCFH-DA (15 min of incubation in the dark). After DCFH-DA exposure, the wells were washed twice with PBS and cells were collected following trypsinization, briefly centrifugated and then resuspended in PBS. Green fluorescence (FL1) was detected on least 5000 cells per sample using a FACScan equipped with a 488 nm argon laser and finally analyzed by employing the CellQuest software (Becton-Dickinson, Milano, Italy).

For comparative purposes HepG2 control cells were treated with 50 µM H_2_O_2_ under normoxic conditions (as positive control).

#### 4.2.9. Lactic Acid Release in Culture Medium

H/3.1 or H/SB3 cells were seeded in 6-well culture plates (106 cells per well), up to 48 h. Lactic acid release in culture medium was evaluated, according the manufacturer’s instructions, by colorimetric assay kit supplied by Biovision Incorporated (155 S Milpitas Blvd. Milpitas, CA 95035, USA).

#### 4.2.10. Measurements of Oxygen Consumption Rate (OCR) and Extracellular Acidification Rate (ECAR)

Cellular metabolic profile was determined using a Seahorse XF24 Extracellular Flux Analyzer (Seahorse Biosciences, USA), providing real-time measurements of oxygen consumption rate (OCR) and extracellular acidification rate (ECAR), as previously described [31]. HepG2 cells transfected with empty vector or over-expressing SB3 were seeded in collagen I coated XF24 cell culture microplates (Seahorse Bioscience), at 1.5 × 10^4^ cells per well. After 24 h, growth medium was removed and replaced with 500 μL of assay medium prewarmed to 37 °C, composed of DMEM without bicarbonate containing 1 mM sodium pyruvate, 2 mM L-glutamine, and left at 37 °C in a CO2-free cell incubator. Measurements of oxygen consumption rate (OCR) and extracellular acidification rate (ECAR) were performed after equilibration in assay medium for 1 h. After an OCR and ECAR baseline measurement, sequential injections through ports in the XF Assay cartridges of pharmacologic inhibitors: Oligomycin (1 mM), an inhibitor of ATP synthase, which allows a measurement of ATP-coupled oxygen consumption through oxidative phosphorylation (OXPHOS); carbonyl cyanide 4-trifluoromethoxy-phenylhydrazone (FCCP) (300 nM), an uncoupling agent that allows maximum electron transport, and therefore a measurement of maximum OXPHOS respiration capacity; and finally Rotenone (2 μM, a mitochondrial complex 1 inhibitor) plus Antimycin (1 μM) were performed and changes in OCR and ECAR were analyzed. 

#### 4.2.11. Proteasome Activity Assay

The in vitro assay of 26S proteasome activities was performed as previously described [55]. Cells or liver samples were collected in lysis buffer (50 mM HEPES, pH 7.5, 150  mM NaCl, 5  mM EDTA, 1% Triton X-100, and 2  mM ATP) without protease inhibitors. Lysate was centrifuged at 10,000 *g* for 10 min at 4  °C. Approximately 15–25  μg of total protein of cell lysates were collected in proteasome activity assay buffer (250  mM HEPES, pH 7.5, 5  mM EDTA, 0.5% NP40, 0.01% SDS, and 2  mM ATP) and were transferred to a UV Flat Bottom 96-well microtiter plate (Thermo Fisher Scientific, Waltham, MA, USA) with 20 µM of the fluorogenic substrate Suc-Leu-Leu-Val-Tyr-AMC (Enzo BML-P802-0005) to measure the caspase-like activity of the proteasome. Free AMC liberated by the substrate hydrolysis was quantified for 2–3 h at 37  °C. Fluorescence (380  nM excitation, 460  nM emission) was monitored using a SpectraMax M2 plate reader (Molecular Devices, CA, USA). Preliminary experiments with control cells indicated that reaction rates were linear for at least 4 h. The data were plotted as Relative Fluorescence Units (RFU).

#### 4.2.12. Cell Cycle Analysis

H/3.1 or H/SB3 cells were seeded in culture plates (5 × 10^5^ cells per well, 35 mm Ø), up to 96 h. At indicated time point cells were trypsinized, centrifugated at 1000 rpm for 10 min and fixed with ethanol (ET-OH 70%), then treated with RNAsi for 30 min (final concentration 0.4 mg/mL) and stained with propidium iodide (final concentration 0.184 mg/mL). The cell cycle was analyzed by flow cytometry and quantified with FCS Express 4 Flow Research Edition software. 

#### 4.2.13. Soft Agar Colony Formation

H/3.1 and H/SB3 cells (10,000/well) were seeded on plates coated with soft agar (40% agarose, 40% Dulbecco’s modified Eagle’s medium (DMEM), 20% FBS). Cells were incubated for 12 days in a 5% CO_2_ atmosphere and then colony formation was evaluated employing phase-contrast microscopy, as previously described [9].

#### 4.2.14. In Vitro Angiogenesis

The angiogenic effect of human recombinant SerpinB3 was assayed by evaluating tube formation with HUVECs seeded on BD Matrigel Matrix Growth Factor Reduced (BD 354230) or BD Matrigel Matrix (as positive control for the technique, BD 354234), as previously described [56], with some modifications. Briefly, 100 µL of Matrigel was added per well of 48-well tissue culture plates and allowed to gel at 37 °C for 30 min. HUVECs (2 × 10^4^ cells/well) were loaded on the top of the Matrigel in medium M199 supplemented with 10% serum, with or without human recombinant VEGF (positive control, 10 ng/mL), SerpinB3 (100 ng/mL) or conditioned medium. After 14 h photographs were taken with digital CCD Hamamatsu ORCA camera linked to an inverted microscope (Leica Microsystems).

### 4.3. Statistical Analysis

Data presented in bar graphs (i.e., for cell culture experiments from average data of at least three independent experiments) represent means ± SEM. Data related to autoluminograms and immunofluorescence images were obtained from at least three experiments providing homologous results. Student’s *t*-test or ANOVA test for analysis of variance were employed to evaluate statistical significance of data (with *p* < 0.05 value considered as significant).

## 5. Conclusions

**Figure 9:**
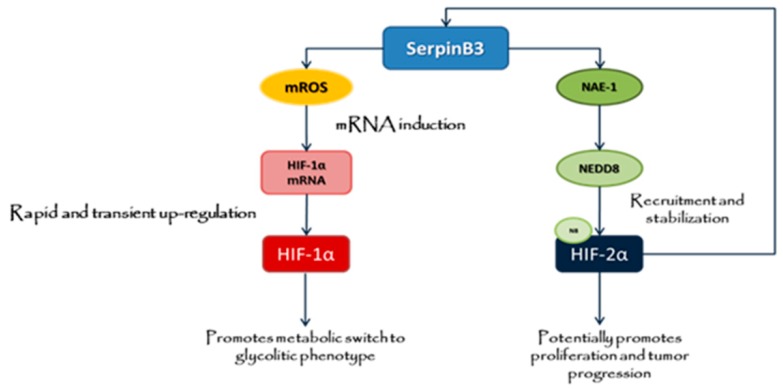
Proposed mechanism and schematic representation of major issue outlined in the present study for SB3-dependent regulation of HIF-1α and HIF-2α. SerpinB3 can affect the behavior of target liver cells by increasing HIF-1α and HIF-2α, with HIF-1α that can act to favor cell survival in a harsh microenvironment by inducing early cellular metabolic switch to glycolytic phenotype, whereas selective NEDDylation and nuclear translocation of HIF-2α can promote proliferation and tumor progression.

In conclusion, we propose that SB3 (Figure 9), whose expression is significantly associated with the worst clinical outcome in HCC patients [37], potentially acts as a paracrine mediator able to affect the behavior of surrounding normal and cancer cells by differently modulating, independently on hypoxic microenvironment, the expression of HIF-1α and HIF-2α proteins, with HIF-1α facilitating cell survival in a harsh microenvironment by inducing early cellular metabolic switch to glycolytic phenotype and HIF-2α potentially promoting proliferation and tumor progression.

In particular, the emerging evidence related to the biological activity of SerpinB3 in tumor microenvironment is of relevance since it outlines two putative novel therapeutic targets involved in cell proliferation and HCC development and progression. Neddylation, a post-translational modification that adds an ubiquitin-like protein NEDD8 to substrate proteins, then affecting their stability as well as their subcellular localization, conformation, and function, has been reported to be over-activated in different human cancers, including lung cancer, intrahepatic cholangiocarcinoma, HCC, colorectal cancer, glioblastoma, nasopharyngeal carcinoma, and esophageal squamous cell carcinoma [31,42,57,58]. Accordingly, targeting the NEDDylation pathway is representing a potential and attractive anticancer therapeutic strategy. Of relevance, the NEDD8-activating enzyme (NAE) inhibitor MLN4924 has been demonstrated to exhibit potent antitumor activity and to be well-tolerated in preclinical studies [34,42,59]. Actually, MLN4924 entered into phase I/II/III clinical trials for patients suffering from solid tumors other than HCC as well as hematologic malignancies [42]. In particular, 33 clinical trials have been enrolled in clinicaltrials.gov website (https://www.clinicaltrials.gov/), and eight completed phase I clinical trials demonstrated that MLN4924 is safe and feasible. Concerning HCC, a recent study from Yang and colleagues has reported a correlation between NAE1 expression and histologic grade, tumor size as well as clinical stage of HCC in patients. Moreover, they showed that the overall survival rate was significantly lower in HCC patients with higher expression of NEDD8 or NAE1, highlighting an association with poor patient prognosis [60].

On the other hand, the intra-tumor hypoxic microenvironment is a condition associated with increased malignancy, poor prognosis, and resistance to clinical treatment (radiotherapy and chemotherapy) [36,61]. However, divergent observations have arisen in separate analysis of some human cancers about the prognostic significant of HIF-1α and HIF-2α; for example, HIF-1α expression has been associated to either better or worst prognosis, depending on tumor type (i.e., renal and non-small cell lung cancers) [36]. Concerning HCC, growing evidence shows a correlation between HIF-2α expression and clinic-pathological features, including tumor grade, venous invasion, necrosis, intrahepatic metastasis, and capsule infiltration, with a significant increase of the overall survival rates of patients with HIF-2α-negative tumors. Accordingly, in a previous study we provided data indicating that in HCC specimens the paired expression of SerpinB3 and HIF-2α transcript levels was significantly correlated, with high levels of both transcripts being detected in HCC specimens from patients that experienced early HCC recurrence. Furthermore, a multivariate analysis with Cox’s regression indicate HIF-2α as an independent significant prognostic factor [61]. Along these lines, perturbation of HIF-2α levels in HCC may represent a novel molecular-targeted therapy for HCC. Actually, PT2385 is a first-in-class, selective small-molecule inhibitor of HIF-2α able to act by blocking HIF-2α/ARNT heterodimerization and inhibiting HIF-2α target genes expression. Preclinical results on a mouse xenograft model of clear cell Renal Cell Carcinoma (ccRCCs), demonstrated that PT2385 has potent antitumor efficacy in terms of decreased expression of HIF-2α target genes, decreased circulating human VEGF-A protein, and increased tumor cell apoptosis as well as tumor regression [38]. More recently, three additional clinical trials have started on glioblastoma and ccRCCs (https://www.clinicaltrials.gov/) but still no human data are available on the use of PT2385 in relation to HCC. Data in the present study, showing that SerpinB3-dependent increased HIF-2α stabilization through direct NEDDylation, should then encourage to consider both HIF-2α and NAE1 as putative novel targets for a selective therapeutic strategy designed to interfere with HCC progression.

## Figures and Tables

**Table 1:** Primers for quantitative real-time PCR (Q-PCR).

Primer	Sense	Reverse
Human HK4	5’-CGCCAAGAAGGAGAAGGTAGAGC-3’	5’-AAGTCCCCGACTTCTGAGCC-3’
Human PKM2	5’-GCTGTCTGGAGAAACAGCCA-3’	5’-GTGACTTGAGGCTCGCACAA-3’
Human HK2	5’-AAATGGAGCGAGGTCTGAGC-3’	5’-CCCACTTCCCATTCCGAACA-3’
Human PDK3	5′-ATTCAATGCCAAAGCGCCAG-3′	AGCAGGGTAGCCCTCTTTT-3′
Human LDHA	5′-CATGGCCTGTGCCATCAGTA-3′	GAAAAGGCTGCCATGTTGG-3′
Human EPO	5′-GAGCCCAGAAGGAAGCCATC-3′	CGGAAAGTGTCAGCAGTGA-3
Humman CXCR4	5′-TCCATTCCTTTGCCTCTTTTGC-3′	CGGAAACAGGGTTCCTTCAT-3′
Human MMP9	5’-TTCAGGGAGACGCCCATTTC-3’	AAACCGAGTTGGAACCACGA-3’
Human NAE-1	5’-GCAAACAAAAACAAATGAAGCCAG-3’	5’-TTTCAGTTCCTGTGGCTGTTG-3’
Murine NAE-1	5’-AGCGGAGAAGATGCTGGAAAC-3’	5’-CTGGACTCTCTTCCACAAAACT-3’
Murine HIF-1α	5’-TCAAGTCAGCAACGTGGAAG-3’	5’-TATCGAGGCTGTGTCGACTG-3’
Murine HIF-2α	5’-CTAAGTGGCCTGTGGGTGAT-3’	5’-GTGTCTTGGAAGGCTTGCTC-3’
Human HIF-1α	5’-CCACCTATGACCTGCTTGGT-3’	5’-TATCCAGGCTGTGTCGACTG-3’
Human HIF-2α	5’-TTGATGTGGAAACGGATGAA-3’	5’-GGAACCTGCTCTTGCTGTTC-3’
Human HO-1	5’-ATGACACCAAGGACCAGAGC-3’	5’-GTGTAAGGACCCATCGGAGA-3’
Human GAPDH	5′-TGGTATCGTGGAAGGACTCATGAC-3′	5′-ATGCCAGTGAGCTTCCCGTTCAGC-3′
Murine GAPDH	5′-TGGAAAGCTGTGGCGTGAT-3′	5′-TGCTTCACCACCTTCTTGAT-3′

## Supplementary Materials

The following are available online at https://www.mdpi.com/2072-6694/11/12/1933/s1, Figure S1: Expression of SB3, HIF-2α, NAE1, HIF-1α and HO-1 in normal human liver tissue sections; Figure S2A–E: In vivo correlation between SB3, HIF-1α and HO-1; Figure S3A–F: In vivo analysis of SB3, HIF-1α and HO-1 in HCC nodules obtained from mice undergoing DEN/CDAA carcinogenic protocol; Figure S4A–B: NAE1 silencing not affect HIF-1α protein levels; Figure S5: Nuclear translocation of HIF-1α and HIF-2α; Figure S6A,B Silencing of HIF-1α affects OCR; Figure S7A–C: HIF-2α is involved in increased proliferation of H/SB3 cells; Figure S8A,B: HIF-1α and HIF-2α silencing confirmed their specific action on related target genes.
