# Supplementary figures and images for: SerpinB3 Differently Up-Regulates Hypoxia Inducible Factors-1α and -2α in Hepatocellular Carcinoma: Mechanisms Revealing Novel Potential Therapeutic Targets
Source: Cancers (Basel). 2019 Dec 4;11(12):1933. doi: 10.3390/cancers11121933 (PMC6966556; doi:10.3390/cancers11121933)

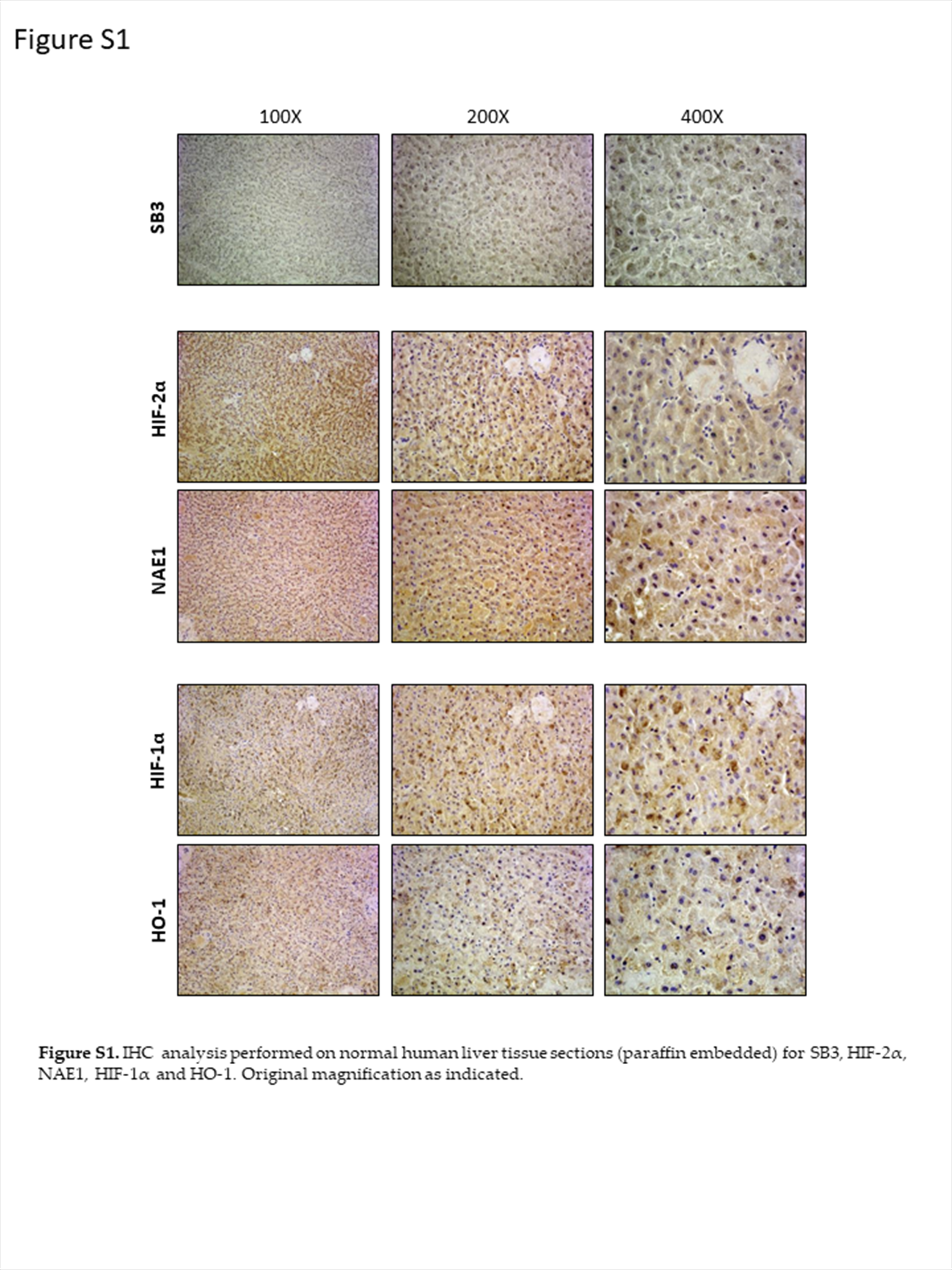

Supplement: Supplementary file 1 [file cancers-11-01933-s001.zip › Figure S 1.tif]

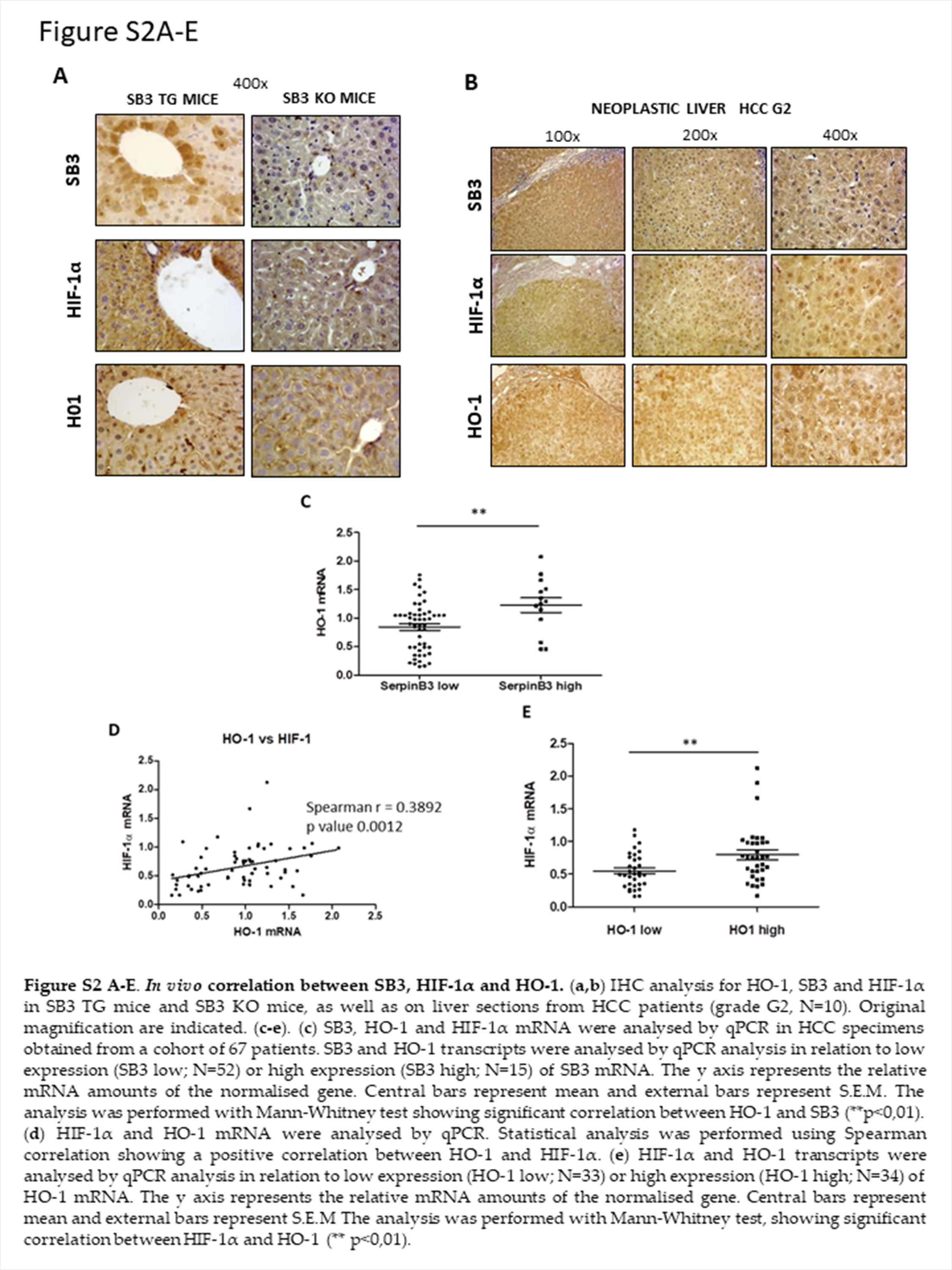

Supplement: Supplementary file 1 [file cancers-11-01933-s001.zip › Figure S 2 A-E.tif]

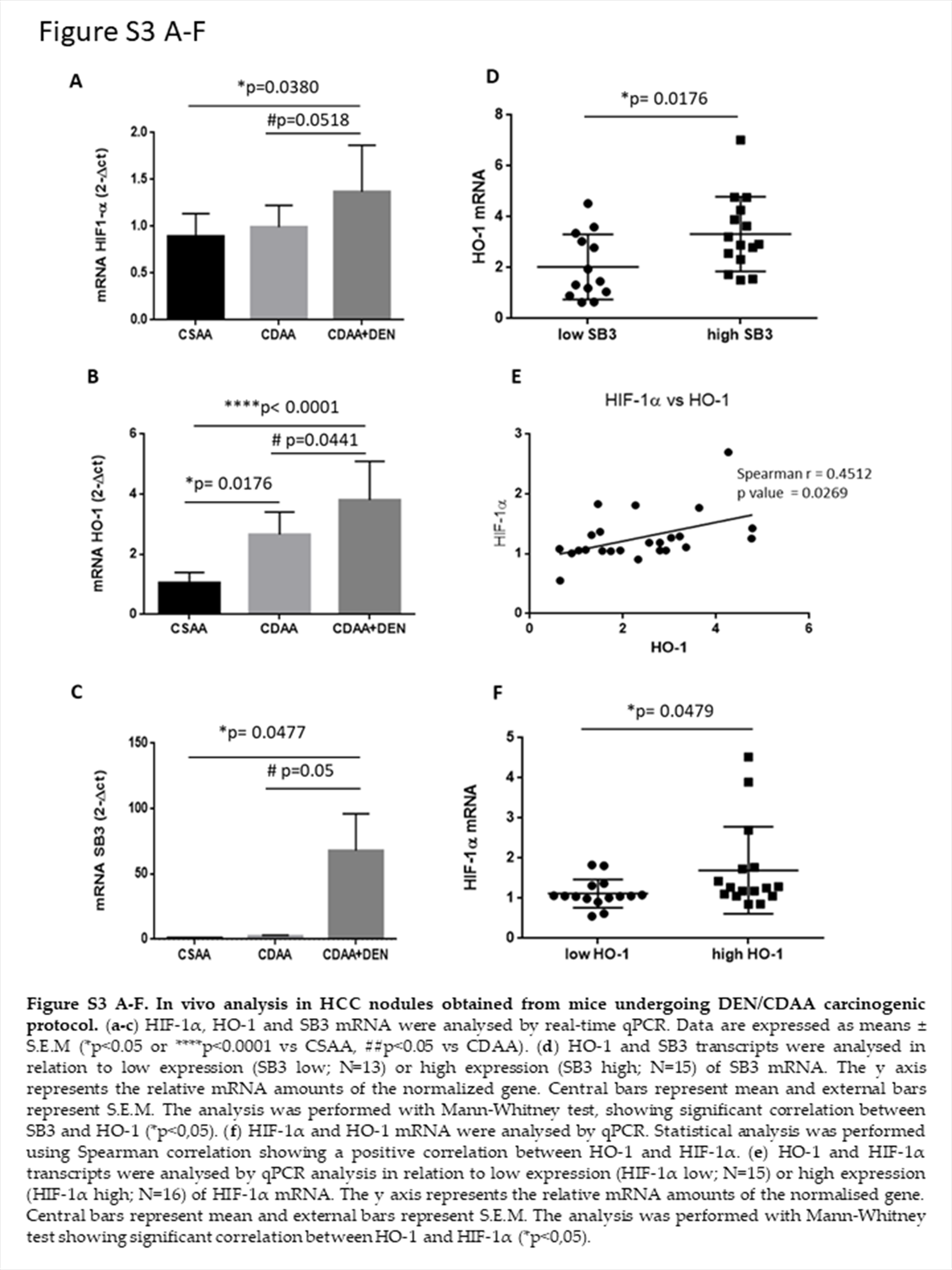

Supplement: Supplementary file 1 [file cancers-11-01933-s001.zip › Figure S 3 A-F.tif]

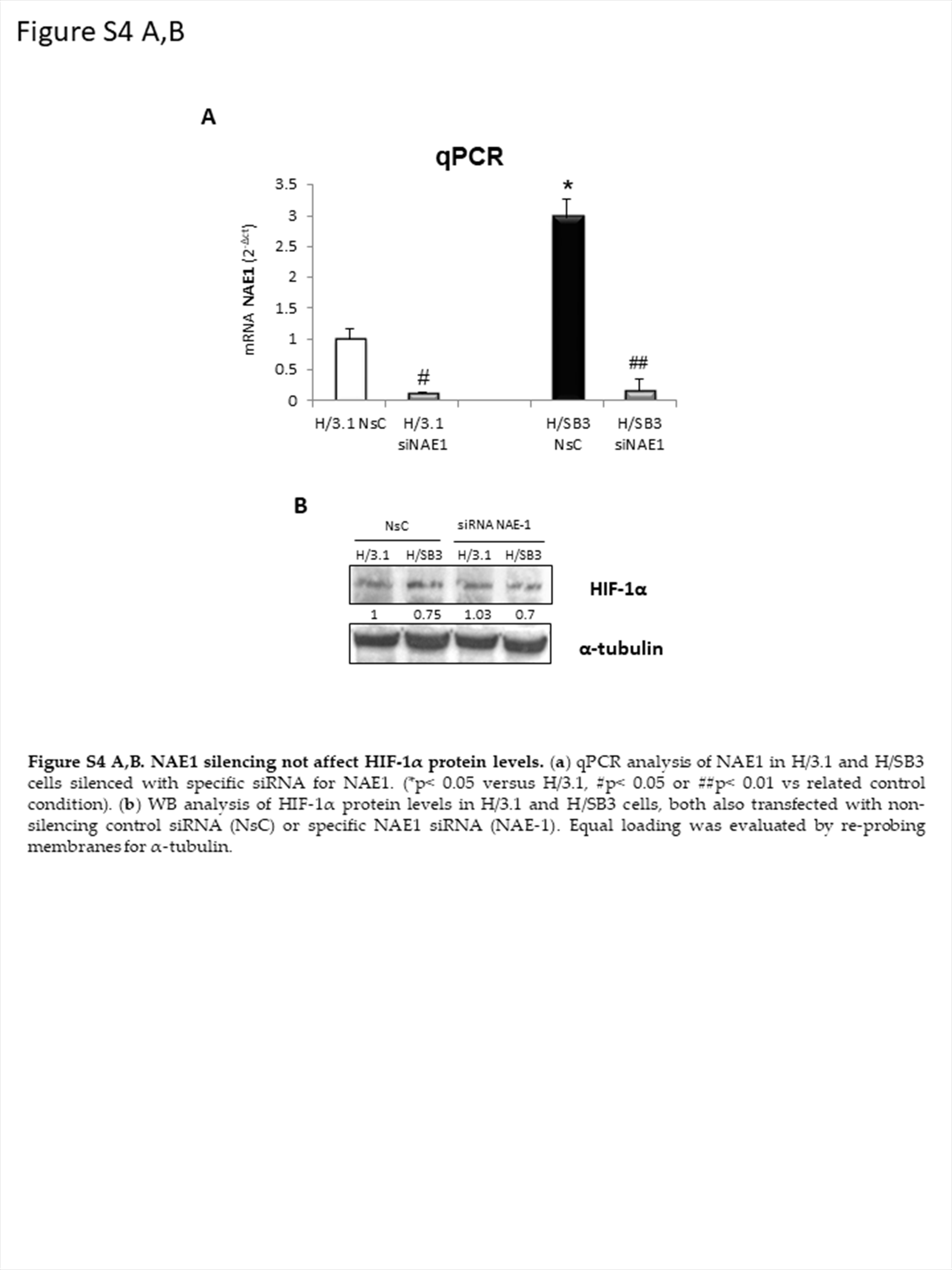

Supplement: Supplementary file 1 [file cancers-11-01933-s001.zip › Figure S 4 A,B.tif]

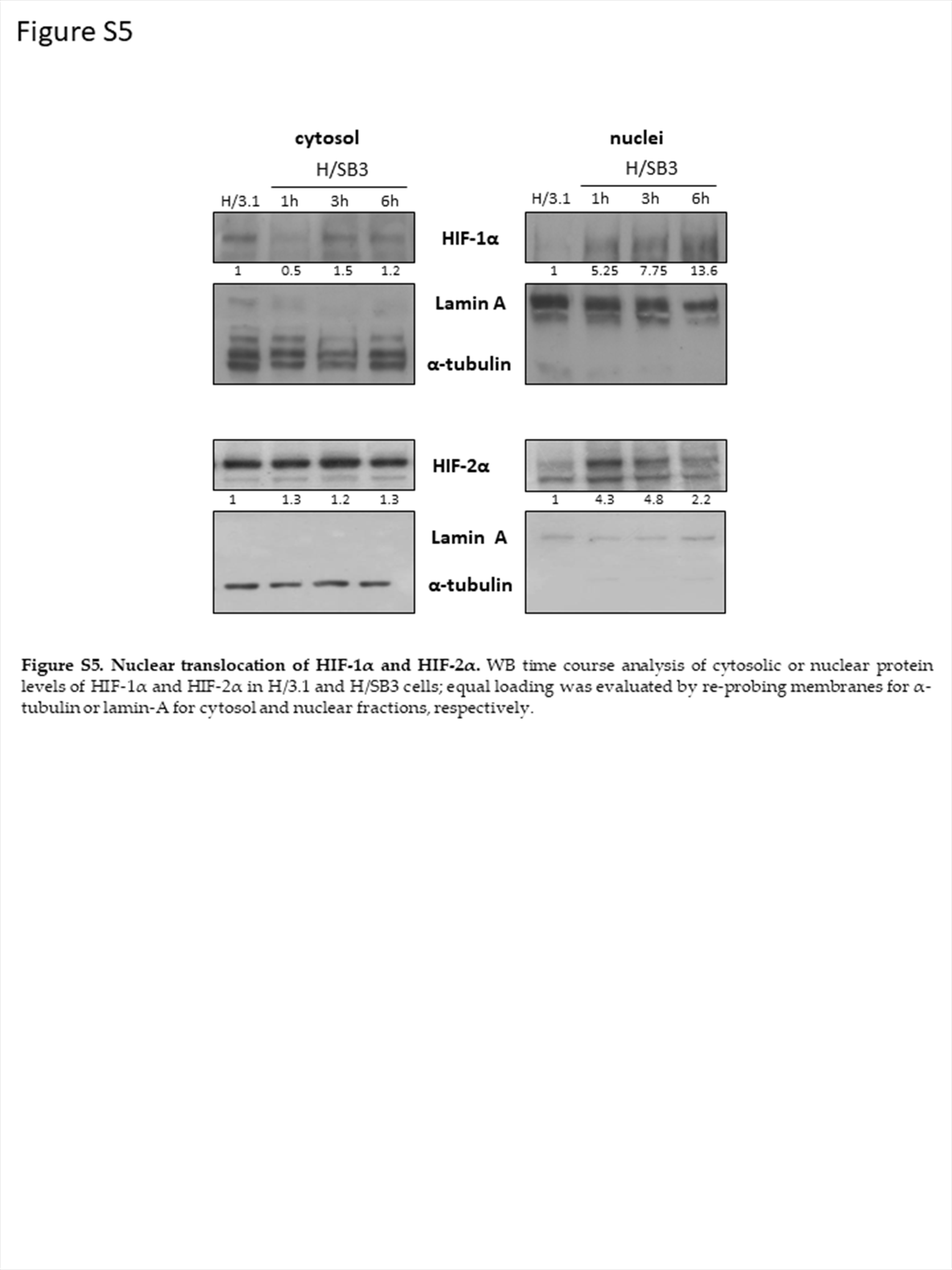

Supplement: Supplementary file 1 [file cancers-11-01933-s001.zip › Figure S 5.tif]

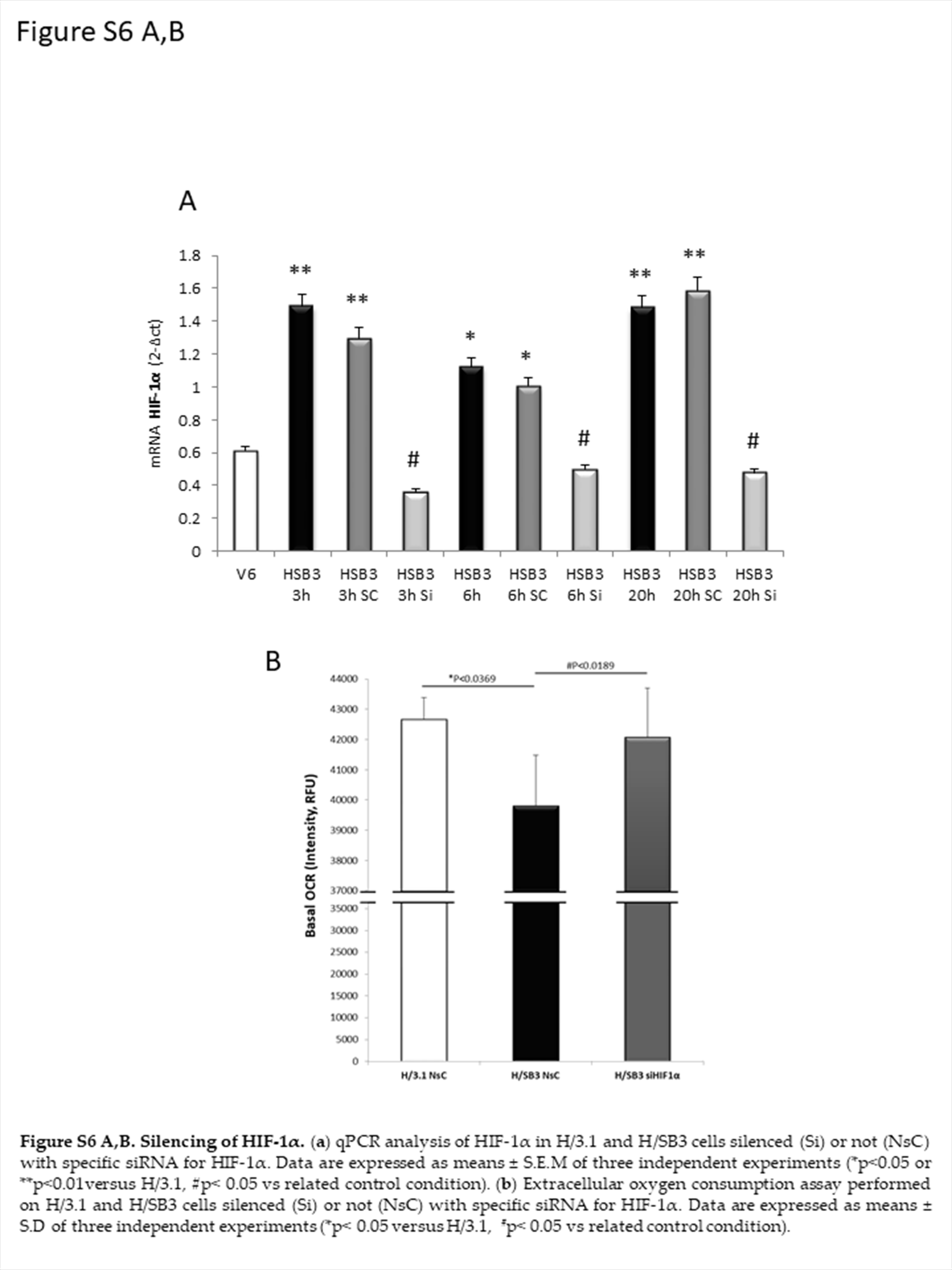

Supplement: Supplementary file 1 [file cancers-11-01933-s001.zip › Figure S 6 A,B.tif]

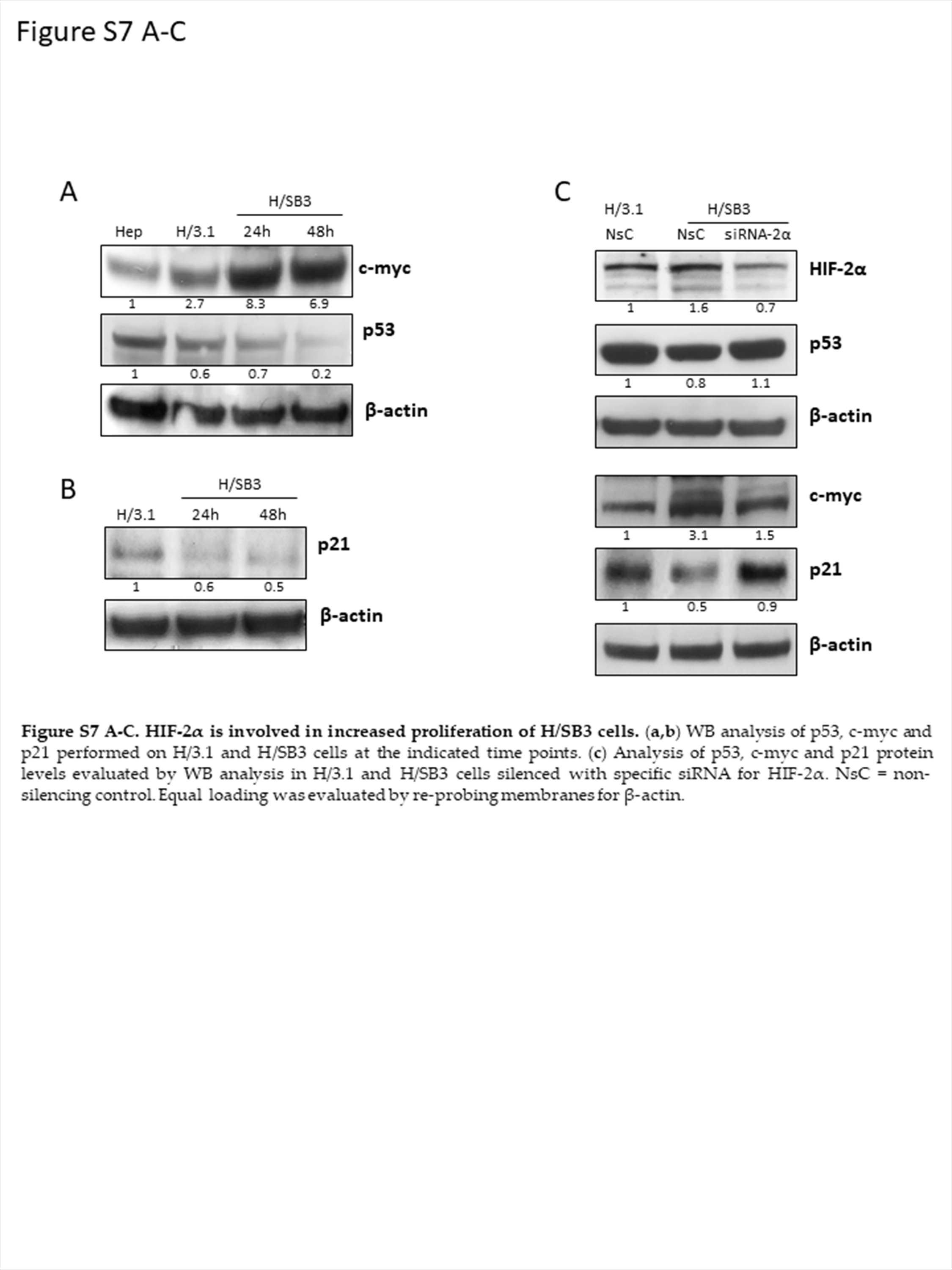

Supplement: Supplementary file 1 [file cancers-11-01933-s001.zip › Figure S 7 A-C.tif]

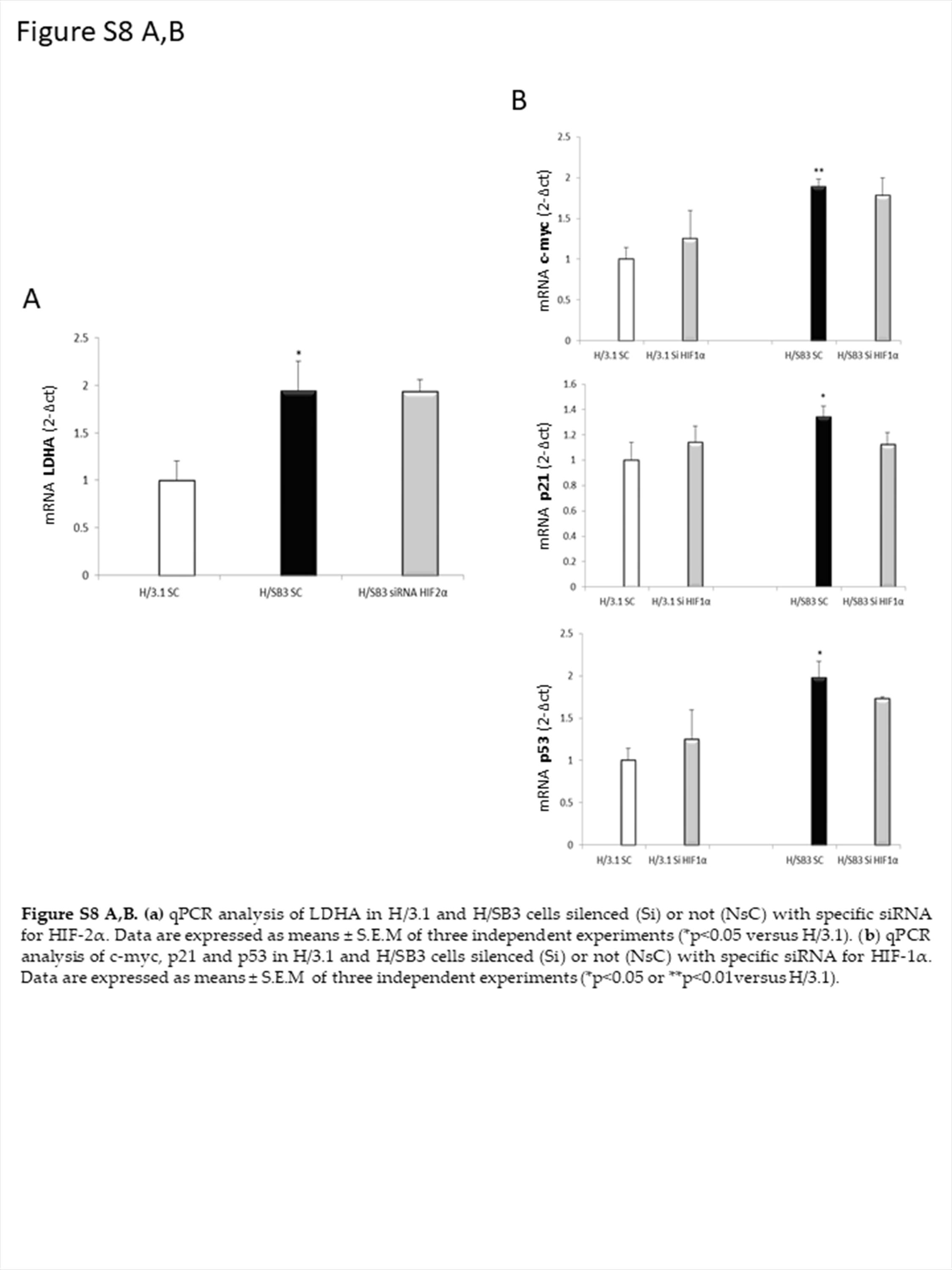

Supplement: Supplementary file 1 [file cancers-11-01933-s001.zip › Figure S 8 A,B.tif]
